# Sugarcane genes associated with sucrose content

**DOI:** 10.1186/1471-2164-10-120

**Published:** 2009-03-21

**Authors:** Flávia S Papini-Terzi, Flávia R Rocha, Ricardo ZN Vêncio, Juliana M Felix, Diana S Branco, Alessandro J Waclawovsky, Luiz EV Del Bem, Carolina G Lembke, Maximiller DL Costa, Milton Y Nishiyama, Renato Vicentini, Michel GA Vincentz, Eugênio C Ulian, Marcelo Menossi, Glaucia M Souza

**Affiliations:** 1 Departamento de Bioquímica, Instituto de Química, Universidade de São Paulo, São Paulo, SP, Brazil; 2 BIOINFO-USP Núcleo de Pesquisas em Bioinformática, Universidade de São Paulo, São Paulo, SP, Brazil; 3 Centro de Biologia Molecular e Engenharia Genética, Universidade Estadual de Campinas, Campinas, SP, Brazil; 4 Departamento de Genética e Evolução, Instituto de Biologia, Universidade Estadual de Campinas, Campinas, SP, Brazil; 5 Centro de Tecnologia Canavieira, Piracicaba, São Paulo, SP, Brazil; 6 Monsanto do Brasil Ltda, São Paulo, SP, Brazil

## Abstract

**Background -:**

Sucrose content is a highly desirable trait in sugarcane as the worldwide demand for cost-effective biofuels surges. Sugarcane cultivars differ in their capacity to accumulate sucrose and breeding programs routinely perform crosses to identify genotypes able to produce more sucrose. Sucrose content in the mature internodes reach around 20% of the culms dry weight. Genotypes in the populations reflect their genetic program and may display contrasting growth, development, and physiology, all of which affect carbohydrate metabolism. Few studies have profiled gene expression related to sugarcane's sugar content. The identification of signal transduction components and transcription factors that might regulate sugar accumulation is highly desirable if we are to improve this characteristic of sugarcane plants.

**Results -:**

We have evaluated thirty genotypes that have different Brix (sugar) levels and identified genes differentially expressed in internodes using cDNA microarrays. These genes were compared to existing gene expression data for sugarcane plants subjected to diverse stress and hormone treatments. The comparisons revealed a strong overlap between the drought and sucrose-content datasets and a limited overlap with ABA signaling. Genes associated with sucrose content were extensively validated by qRT-PCR, which highlighted several protein kinases and transcription factors that are likely to be regulators of sucrose accumulation. The data also indicate that aquaporins, as well as lignin biosynthesis and cell wall metabolism genes, are strongly related to sucrose accumulation. Moreover, sucrose-associated genes were shown to be directly responsive to short term sucrose stimuli, confirming their role in sugar-related pathways.

**Conclusion -:**

Gene expression analysis of sugarcane populations contrasting for sucrose content indicated a possible overlap with drought and cell wall metabolism processes and suggested signaling and transcriptional regulators to be used as molecular markers in breeding programs. Transgenic research is necessary to further clarify the role of the genes and define targets useful for sugarcane improvement programs based on transgenic plants.

## Background

The importance of bioenergy-generating crops such as sugarcane is increasing rapidly and is likely to play an increasing role given the environmental and economical challenges of fossil fuel usage. Sugarcane belongs to the *Saccharum *L. genus, which derives from crosses of the domesticated species *S. officinarum *(a group that has sweet canes with thick and juicy culms), natural hybrids (*S. sinense *and *S. barberi*) and *S. spontaneum *(a wild species with no sugar and thin culms). All modern cultivars are derived from a few intercrossings of these hybrids [1-5]. Sucrose content is a phenotypic characteristic selected over centuries by breeding programs. Sugarcane cultivars differ in both maximum sucrose accumulation capacity and accumulation dynamics during growth [6]. Breeding programs routinely perform crosses to identify genotypes able to produce more sucrose early in the crop season to allow for continuous sugar production throughout the year. The internodes mature progressively towards the base of the culms with an increasing concentration of sucrose at the base. Sucrose content in the mature internodes can reach around 20% of the culms dry weight while lower sucrose levels are observed in younger internodes where glucose and fructose are predominant.

The improvement of modern cultivars could be achieved by identifying genes associated with important agronomic traits, such as sucrose content. These genes can then be used to generate transgenic plants or can serve as molecular markers for map-assisted breeding [7]. Internodes have been expression-profiled during culm development [8-12], but differences between cultivars that contrast for sucrose content have not been extensively reported. Understanding differences in the regulation of genes related directly or indirectly to sucrose accumulation in different cultivars is an important step if we want to aid breeding for sugar yield improvement. It is also important to understand the impact of environmental stresses on sucrose accumulation and the role of hormones in integrating stress signaling and developmental cues. Water stress, for example, reduces yield drastically and therefore, drought-tolerant sugarcane cultivars might be critically important in a scenario of cultivation expansion since much of the land available for sugarcane cultivation is located in regions subjected to drought. Drought responses include immediate protective measures and long term growth alterations [13]. Modulation of gene expression under this stress [14-19] involves ABA-dependent and independent pathways [13]. Carbohydrate metabolism is also related to abiotic stress responses since some aspects of the regulation of sugar metabolism are mediated by ABA and fructose, raffinose and trehalose act as osmoprotectants [20]. It is important to emphasize that some sugars (such as glucose, trehalose and sucrose) are important signaling molecules that affect plant growth and development including germination, early vegetative growth and flowering, as well as a variety of physiological processes such as photosynthesis, resource partition and defense responses [21-26]. The pathways activated by sugars cross-talk with other pathways, including those related to hormonal, cell cycle control and nitrogen responses [27-30]. ABA and sucrose were shown to be involved in the control of sucrose levels in plant cells [21] but the underlying mechanisms are still unknown.

We previously used cDNA microarrays to identify sugarcane genes that are responsive to drought and ABA [31]. The cDNAs are derived from a collection of 237,954 ESTs developed by the SUCEST sugarcane EST project [32] which were assembled into 43,141 putative, unique sugarcane transcripts that are referred to as Sugarcane Assembled Sequences (SAS). In this report we present the results of a large-scale analysis of the transcriptome of thirty genotypes grown in the field. cDNA microarrays were used to compare high- and low-Brix individuals and a comparison was made to reveal gene expression patterns that correlate with sucrose content, culm development, sugar treatments, drought and ABA treatment. We performed an extensive validation of cDNA microarray data using pooled plants, as well as individual genotypes. The results indicate a close relationship between sucrose content and drought signaling.

## Results

cDNA microarrays were used to identify genes that were differentially expressed in genotypes contrasting for sucrose content. The arrays preparation, validation and analysis were done as previously described [31]. Multiple crossings were performed for twelve years among *S. officinarum *and *S. spontaneum *(Population 1) and between commercial varieties SP80-180 and SP80-4966 (Population 2) to generate genotypes with extreme values of sugar content. The simplest way to access phenotypic differences with a high degree of confidence is to measure sucrose in the culm juice. This can be done in the field using a simple refractometer that evaluates Brix (soluble solids content). In sugarcane most of the soluble solids in the juice (70 to 91%) correspond to sucrose. Using this approach, thousands of genotypes can be phenotyped and contrasting individuals among the populations can be selected for further agronomic evaluation. Brix measurements were taken from 500 individuals of each population and the extreme clones in this population were selected and evaluated for sucrose content (see Additional file 1). To evaluate gene expression samples were collected from single individuals as well as from pools of seven or eight plants grown for seven, ten and elevenmonths.

**Figure 1:**
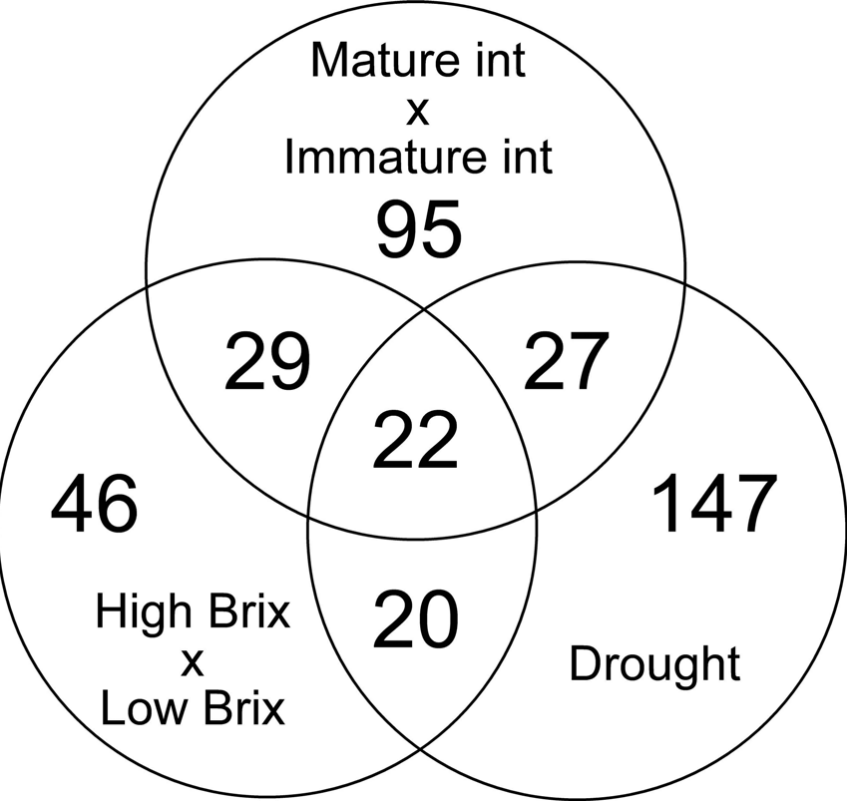
**Comparison of differential gene expression associated with sucrose content, culm development and drought responses in sugarcane**. Genes were identified as associated with sucrose content if they were differentially expressed when high Brix or low Brix pools of plants were compared. Genes regulated during culm development were identified by comparing Mature and Immature Internodes. The drought-responsive genes were found to be induced or repressed by drought after 24, 72 or 120 h of water deficit. The figure represents a Venn diagram of the three differential expression data sets. Technical replicates range from 2 to 16 since genes are spotted several times in the same array. The credibility level used to define outliers was 0.96 in all three data sets.

Two experimental designs were used to perform transcriptome comparisons: (I), internodes 1, 5 and 9 from high Brix plants were compared to the same internodes from low Brix plants (HB vs LB) in both populations or (II), mature internodes 9 were compared to immature internodes 1 from plants with high or low Brix in population 2 [33]. Twenty six hybridizations were performed revealing 239 genes associated with sucrose content and regulated during culm development (see Additional file 2 and Figure 1).

A total of 117 genes were found to be differentially expressed in at least one comparison between high and low Brix genotypes (internodes 1, 5 or 9), and ten genes (SCCCLR1048F03.g, SCCCLR2003E10.g, SCCCRZ1001F02.g, SCCCRZ1001H05.g, SCCCRZ1002E08.g, SCEZST3147A10.g, SCJFRZ2007F10.g, SCAGLR1043E04.g, SCSBHR1050B11.g and SCVPCL6041F01.g) were found to be differentially expressed in both populations analyzed (see Additional file 2). Among these SAS, we found three transcription factors, two aquaporins and two transcripts related to development. The gene expression comparison between mature and immature internodes showed a total of 173 differentially expressed genes (see Additional file 2 and Figure 1).

Table 1 lists a selection of the differentially expressed genes along with the number of biological samples that displayed altered expression when high and low Brix pools of plants were compared (HB vs LB) and when mature and immature internodes were compared (MI vs II). The expression data sets were compared to those obtained for plants exposed to drought conditions or ABA treatment [31] (see Additional file 2). Comparison to ABA treated plants yielded eleven differentially expressed genes in common, including the *ScPKABA1-3 *(SCRFLR1034G06.g) and the *ScMAPK-4 *(SCSBAM1084E01.g), which were both more expressed in high Brix and repressed by ABA, and a PP2C-like protein phosphatase (SCEPRZ1010E06.g) which showed the opposite profile. Comparison to drought-regulated genes showed an extensive overlap in differential expression between the two datasets. Between 117 and 173 genes associated with high sucrose content and internode development, respectively, 43.6% and 28.3% were previously shown to be altered by drought while twenty-two genes were altered in all conditions analyzed (Figure 1).

**Table 1:** Selection of SAS showing differential expression when high and low Brix plants were compared or when mature and immature internodes were compared.

**SAS**	**category**	**sub category 1**	**sub category 2**	**HB vs LB**	**MI vs II**	**Drought**	**ABA**	**Suc**	**Gluc**
SCCCLR1022D05.g	adapter	14-3-3 protein	GF14		↓				
SCCCRZ1001D02.g	adapter	14-3-3 protein	GF14		↓↓↓↓				
SCEQRT1031D02.g	adapter	14-3-3 protein	GF14		↓↓				
SCEQRT1025D06.g	adapter	14-3-3 protein	GF14		↓	↓↓			
SCVPLR1049C09.g	calcium metabolism	calmodulin-binding protein	AAA family ATPase (cell division cycle protein 48 sub-family)		↓				
SCCCRZ1C01H06.g	calcium metabolism	calmodulin-binding protein	Apyrase (Nucleoside diphosphatase)		↓↓↓↓	↓↓			
SCJLLR1108H07.g	calcium metabolism	calmodulin-binding protein	Ca(2+)-ATPase		↓				
SCEZLB1012F10.g	calcium metabolism	calmodulin-binding protein	Cyclic nucleotide-gated calmodulin-binding ion channel	↑					
SCCCAM1001A03.g	calcium metabolism	calmodulin-binding protein	Multidrug resistant (MDR) ABC transporter	↑				↑↑	
SCRFLR2037F09.g	calcium metabolism	calreticulin	CRT2 Calreticulin 2	↓	↓↓	↑↑		↑↑↑	↑↑↑
SCCCLR2C02A05.g	cell wall metabolism	expansin	EXPA11	↓	↓↓				
SCQGRT1040G03.g	cell wall metabolism	expansin	OsEXPA23	↑		↓↓			
SCACSB1037A07.g	cell wall metabolism	cytochrome P450	P-coumaroyl shikimate 3'-hydroxylase	↓↓					
SCEZHR1087F06.g	cell wall metabolism	cytochrome P450	Ferulate-5-hydroxylase	↓	↑↑				
SCSGFL4193B05.g	cell wall metabolism	cytochrome P450	Cinnamic acid 4-hydroxylase	↓					
SCRFLR1012F12.g	cell wall metabolism	.	Caffeic acid 3-O-methyltransferase	↑↑	↑↑				
SCBFLR1039B05.g	cell wall metabolism	polysaccharide metabolism	Xyloglucan endotransglycosylase		↓↓↓↓				
SCCCLR1048D07.g	cell wall metabolism	lignin	Phenylalanine ammonia-lyase	↑		↓↓			
SCEQRT1024E12.g	cell wall metabolism	lignin	Phenylalanine ammonia-lyase	↑	↓	↓↓	↑↑	↑↑↑	↑↑↑
SCSGAM1094D05.g	cell wall metabolism	lignin	Phenylalanine ammonia-lyase	↓	↓				
SCCCCL6002B05.g	hormone biosynthesis	auxin	Nitrilase	↑		↑↑			
SCEQRT1028H06.g	hormone biosynthesis	auxin	Nitrilase		↓↓	↑↑			
SCRFLR1012D12.g	hormone biosynthesis	auxin	Nitrilase	↑	↓	↑↑			
SCVPLR2012A10.g	hormone biosynthesis	ethylene	ACC oxidase	↑	↓↓				
SCCCRT1001E01.g	hormone biosynthesis	jasmonic acid	Lipoxygenase	↓	↓↓↓↓	↓↓		↓↓↓	↓↓↓
SCJFRT1007H07.g	hormone biosynthesis	jasmonic acid	Lipoxygenase	↓	↓				
SCCCLR1C03G01.g	hormone biosynthesis	jasmonic acid	Omega-6 fatty acid desaturase	↓	↓	↑↑	↑↑		
SCCCAM2004G02.g	hormone-related	auxin	Auxin transport/auxin eflux carrier (OsPIN1c)	↓					
SCCCLR2002F08.g	hormone-related	auxin	dormancy/auxin associated family (auxin-repressed)	↓	↑↑				
SCBGLR1023D05.g	pathogenicity	R-gene transduction	Zinc finger protein (LSD1)	↑	↓↓↓	↓↓		↑↑↑	
SCAGLR1043F02.g	protein metabolism	calmodulin-binding protein	HSP70 (heat shock)	↑↑	↓	↑↑			
SCCCCL3120G07.g	protein metabolism	calmodulin-binding protein	HSP70 (heat shock)	↑		↑↑			
SCCCRZ1003A03.g	protein metabolism	calmodulin-binding protein	HSP70 (heat shock)		↑				
SCEQRT2099H01.g	protein kinase	calcium-dependent	ScCDPK-27		↓				
SCVPAM1055A12.g	protein kinase	casein kinase	ScCKI-11	↑	↓	↑↑			
SCCCLR1C04G08.g	protein kinase	casein kinase	ScCKI-3	↑					
SCCCLR1022H07.g	protein kinase	cell cycle-related	ScCDK-11		↓				
SCBGLR1096C08.g	protein kinase	cell cycle-related	ScCDK-18		↓				
SCVPRT2081G05.g	protein kinase	cell cycle-related	ScCDK-3		↓				
SCRLFL1012B10.g	protein kinase	cell cycle-related	ScCDK-6		↓				
SCSBAM1084E01.g	protein kinase	MAPK/MAPKK/MAPKKK	ScMAPK-4	↑	↑↑		↓↓		
SCEPAM1020A03.g	protein kinase	other	ScATN1-2	↓					
SCVPCL6042B07.g	protein kinase	other	ScCyclin G-associated kinase-like protein-1		↓				
SCJFRZ2032C08.g	protein kinase	SNF-like kinase	ScCIPK-14		↑	↑↑			
SCBFSB1046D04.g	protein kinase	SNF-like kinase	ScCIPK-16	↑				↓↓↓	
SCMCRT2103B04.g	protein kinase	SNF-like kinase	ScCIPK-21	↑↑	↑	↓↓		↓↓	
SCCCLR1C05B07.g	protein kinase	SNF-like kinase	ScCIPK-3	↑		↑↑		↓↓↓	↓↓↓
SCJLRZ1023H04.g	protein kinase	SNF-like kinase	ScCIPK-9		↓↓	↓↓			
SCEPRZ1009C10.g	protein kinase	SNF-like kinase	ScOSA PK-1		↓↓			↓↓	↓↓
SCCCST1004A07.g	protein kinase	SNF-like kinase	ScOSA PK-7		↓				
SCACLR2007G02.g	protein kinase	SNF-like kinase	ScPKABA1-1	↑↑				↓↓	↓↓↓
SCRFLR1034G06.g	protein kinase	SNF-like kinase	ScPKABA1-3	↑			↓↓	↓↓↓	↓↓↓
SCJFRZ2032G01.g	protein kinase	SNF-like kinase	ScSnRK1-2		↓↓	↓↓		↑	
SCCCCL5002B10.g	protein kinase	undefined	ScPK-BI2		↓↓↓				
SCJLLR1054C03.g	protein kinase	undefined	ScPK-BIII7	↑					
SCMCSD2061D05.g	protein kinase	undefined unclassified	ScUPK-46 (CIPK)	↓					
SCCCLB1001D03.g	protein phosphatase	serine/threonine PPM family	PP2A/Catalytic Subunit		↓				
SCEZLR1052F07.g	protein phosphatase	serine/threonine PPM family	PP2A/Subunit A		↓				
SCEPRZ1010E06.g	protein phosphatase	serine/threonine PPM family	PP2C-like	↓↓	↓	↑↑	↑↑	↓↓↓	↓↓↓
SCEZHR1088E02.g	protein phosphatase	tyrosine phosphatase	Dual Specificity Protein Phosphatases (DSPP)	↑↑↑	↓	↑↑		↓↓↓	↓↓↓
SCMCST1051F08.g	protein phosphatase	tyrosine phosphatase	Tyrosine Specific Protein Phosphatases (PTP)			↓↓			
SCSBHR1056H08.g	receptor	ethylene	EIN2		↑				
SCUTLR2023D06.g	transcription factor	CCAAT	ScCA2P5	↑					
SCCCLR1066G08.g	transcription factor	HGM (high mobility group protein)		↑		↓↓			
SCBFAD1046D01.g	transcription factor	HLH (helix-loop-helix)	ScbHLH1		↓↓				
SCCCRZ1001H05.g	transcription factor	HLH (helix-loop-helix)	ScbHLH7	↑↑		↓↓		↓↓↓	↓↓↓
SCAGLR1021G10.g	transcription factor	homeobox	ScHB2		↓↓			↓↓↓	↓↓↓
SCRLAM1010D08.g	transcription factor	homeobox	ScHB41		↓↓				
SCEZLB1010E10.g	transcription factor	hormone-related/auxin	ScABI40	↓					
SCCCLR1024F10.g	transcription factor	hormone-related/auxin	ScARF46		↓				
SCCCRZ1001G10.g	transcription factor	hormone-related/Aux/IAA	ScAUXI134		↓↓↓↓	↓↓		↓↓↓	↓↓↓
SCVPLR2005H03.g	transcription factor	hormone-related/Aux/IAA			↓↓				
SCJFRZ2009F04.g	factor transcription	hormone-related/Aux/IAA			↓				
SCJLLR1054C09.g	transcription factor	hormone-related/Aux/IAA			↓↓				
SCUTST3086B02.g	transcription factor	hormone-related/ethylene/AP2/ERE BP	ScEREB59		↓				
SCCCLR1001D10.g	transcription factor	hormone-related/ethylene/AP2/ERE BP	DRE binding factor 2		↑	↑↑			
SCBGFL4052C11.g	transcription factor	hormone-related/ethylene	ScEIL1		↓				
SCCCRZ1004H12.g	transcription factor	hormone-related/ethylene	ScEIL2	↓	↓				
SCCCRZ2C03D11.g	transcription factor	hormone-related/gibberellin	ScGRAS71		↓↓				
SCEPRZ1008F02.g	transcription factor	LIM (protein-protein interaction)		↓	↓↓				
SCQGLR1085G10.g	transcription factor	MADS	ScMADS17		↑	↓↓			
SCSFAD1124E07.g	transcription factor	MYB	ScMYB70		↑				
SCRURT2010A10.g	transcription factor	MYB	ScMYB120		↓				
SCCCLR2003E10.g	transcription factor	NAM (no apical meristem)	ScNAC27	↓↓				↓↓↓	↓↓↓
SCRUAD1132D09.g	transcription factor	NAM (no apical meristem)	ScNAC51						
SCACLR1130H08.g	transcription factor	zinc finger protein	ScYAB16		↓				
SCEZST3147A10.g	transcription factor	zinc finger protein	ScC3H84	↓	↓				
SCCCCL4003D08.g	transcription factor	zinc finger protein	ScC3H95		↓				
SCQGRZ3011D06.g	transcription factor	zinc finger protein/alfin-like	ScALF9		↓				
SCCCRZ1002E08.g	stress	drought and cold response	Aquaporin (plasma membrane)	↓	↓↓				
SCCCST3001H12.g	stress	drought and cold response	Aquaporin (plasma membrane)	↑	↓↓				
SCEQRT2100B02.g	stress	drought and cold response	Aquaporin (plasma membrane)	↑	↓↓				
SCCCLR1024C03.g	stress	drought and cold response	Aquaporin (tonoplast intrinsic protein)	↓	↓				
SCCCRZ1001F02.g	stress	drought and cold response	Aquaporin (tonoplast intrinsic protein)	↓	↓				
SCQGLR1085F11.g	stress	drought-induced	Dehydrin	↓	↓↓↓	↑↑		↓↓↓	↓↓↓
SCCCLR2C01F06.g	stress	wound-induced	wound-responsive family protein	↑↑↑	↑			↑↑↑	↑↑↑

The table also shows differential expression of the same SAS as seen in [31] for plants submitted to drought and ABA treatment. Differential expression refers to cDNA microarray analysis except for the last two columns, which refer to qRT-PCR data obtained in samples of plantlets treated with sucrose or glucose. The table lists a selection of SAS whose expression was enriched or decreased in two technical replicates for each biological sample. For a complete list see additional file 2. The up arrow indicates that the SAS is more expressed, the down arrow indicates that the SAS is less expressed. The number of arrows indicates the number of hybridizations.

**Figure 2:**
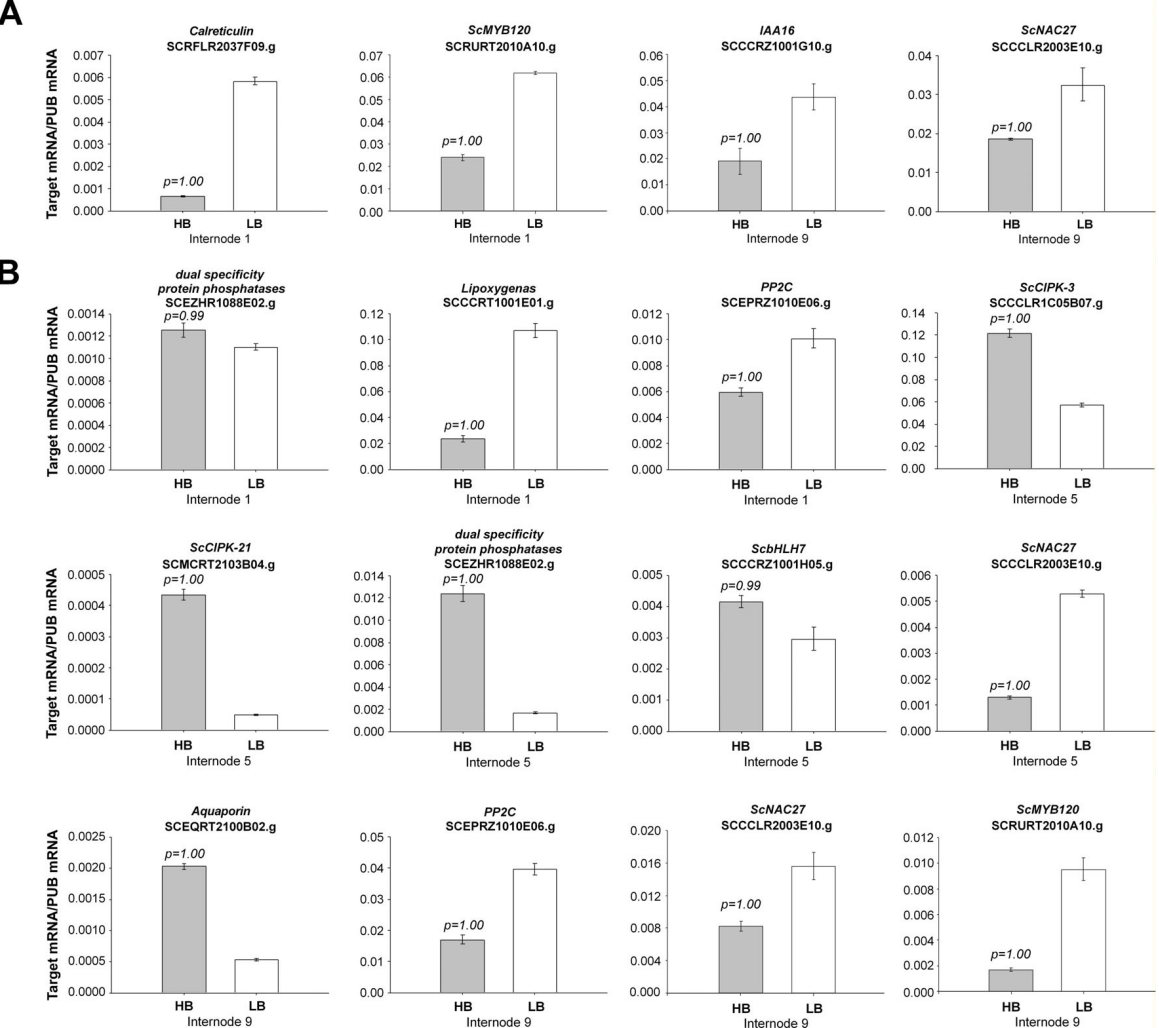
**Real Time PCR (qRT-PCR) analysis of Populations gene expression**. The y axis refers to the relative expression ratio between target mRNA versus the reference mRNA (polyubiquitin-PUB SCCCST2001G02.g). The relative expression levels were determined in Internode 1, 5 and 9 tissues from a pool of the eight individuals with the highest Brix measures (HB) and the eight individuals with the lowest Brix measures (LB) from Population 1 (A) and from a pool of the seven individuals with the highest Brix measures (HB) and the seven individuals with the lowest Brix measures (LB) from Population 2 (B). The reactions for the target mRNA and reference mRNA were carried out in parallel and each reaction was performed in triplicates. Error bars were calculated as described previously [31]. The transcript levels for the reference genes were verified not to vary in response to the treatments. The values of P correspond to the probability Pr(HB>LB) and Pr(HB<LB) for up- and down-regulated genes, respectively. The SAS was considered differentially expressed when P ≥ 0.95.

**Figure 3:**
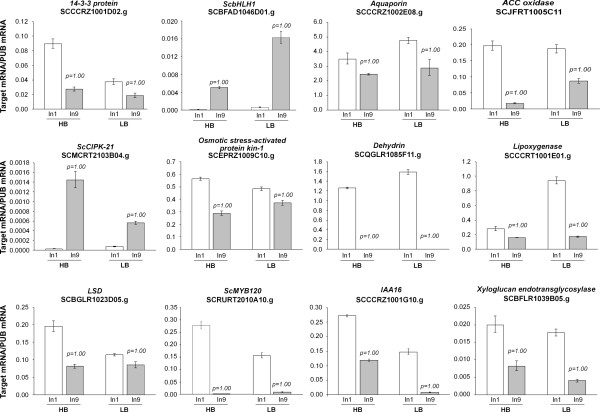
**Real Time PCR (qRT-PCR) analysis of internode developmental gene expression**. The y axis refers to the relative expression ratio between target mRNA versus the reference mRNA (polyubiquitin SCCCST2001G02.g). The relative expression levels were determined in Internode 1 and 9 tissues from a pool of the seven individuals with the highest Brix measures (HB) and the seven individuals with the lowest Brix measures (LB) of Population 2. All reactions were carried out in parallel and each reaction was performed in triplicates. Error bars were calculated as described previously [31]. The transcript levels for the reference genes were verified to not vary in response to the treatments. The P values correspond to the probability Pr(MI>II) and Pr(MI<II) for up- and down-regulated genes, respectively when In9 and In1 samples were compared. The values of P were calculated for the HB and LB pools of plants independently. The SAS was considered differentially expressed when P ≥ 0.95.

**Figure 4:**
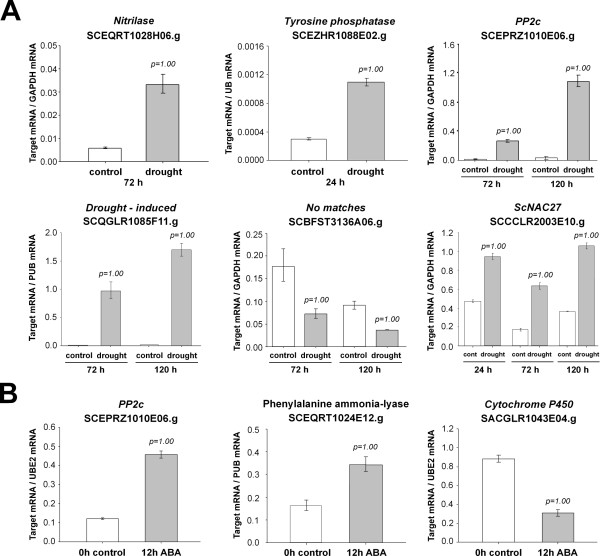
**Real Time PCR (qRT-PCR) analysis of drought and ABA-responsive gene expression**. The y axis refers to the relative expression ratio between target mRNA versus the reference mRNA (polyubiquitin SCCCST2001G02.g; GAPDH Gene ID: 542367; UBE2 SCBGLR1002D06.g) in sugarcane plants treated with ABA for 12 h or drought conditions for 24, 72 or 120 h. The reactions for the target mRNA and reference mRNA were carried out in parallel and each reaction was performed in triplicates. Error bars were calculated as described previously [31]. The transcript levels for the reference genes were verified to not vary in response to the treatments. The values of P correspond to the probability Pr (Treated>Control) and Pr (Treated<Control) for up- and down-regulated genes, respectively. The SAS was considered differentially expressed when P ≥ 0.95.

**Figure 5:**
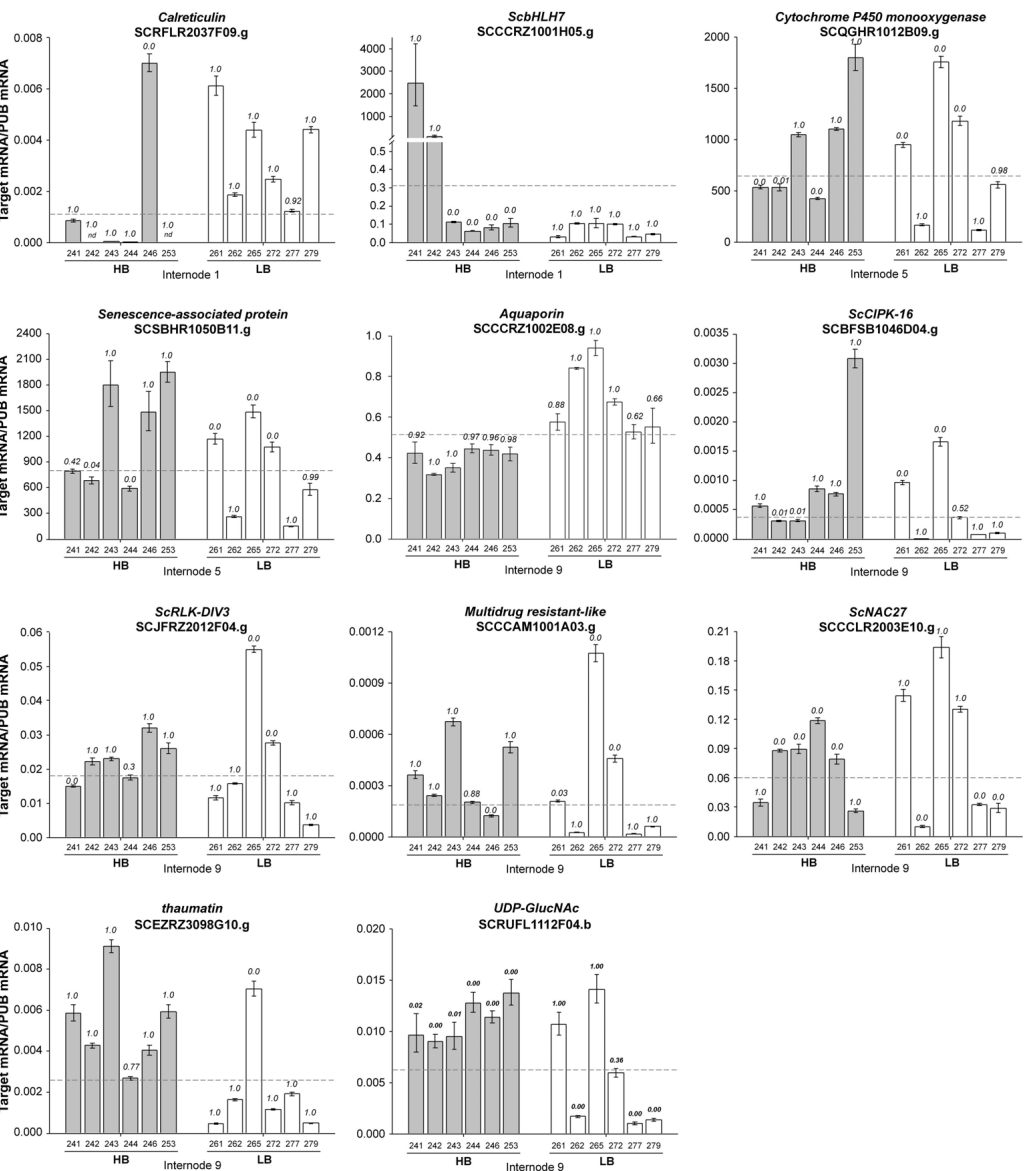
**Real Time PCR (qRT-PCR) analysis of individual genotypes gene expression**. The y axis refers to the relative expression ratio between target mRNA versus the reference mRNA (polyubiquitin SCCCST2001G02.g). The relative expression levels were determined in Internode 1, 5 and 9 tissues from six individuals with the highest Brix measures (CTC98-241, CTC98-242, CTC98-243, CTC98-244, CTC98-246 and CTC98-253) and six individuals with the lowest Brix measures (CTC98-261, CTC98-262, CTC98-265, CTC98-272, CTC98-277 and CTC98-279) of Population 2. All reactions were carried out in parallel and each reaction was performed in triplicates. Error bars were calculated as described previously [31]. The transcript levels for the reference genes were verified to not vary in response to the treatments. The significance of differential gene expression was determined considering normal distributions for each tested condition and comparing them to the average expression for all samples (dotted line). The values of P correspond to the probability Pr (GenotypeX>average) and Pr (GenotypeX<average) for up- and down-regulated genes. P values were calculated for each genotype independently. The SAS was considered differentially expressed when P ≥ 0.95.

Expression data of forty-two genes was also obtained using qRT-PCR. We determined gene expression differences for pools of extreme individuals from both populations (Figure 2), in mature and immature internodes (Figure 3) and in response to drought and ABA treatment (Figure 4). The significance of the data obtained by qRT-PCR was inferred statistically by calculating values of P for expression differences against the reference sample (see Methods for details). Overall gene expression data obtained using cDNA microarrays was confirmed in qRT-PCR experiments for over 80% of the genes tested, even when the target RNA derived from a distinct biological replicate. We also investigated, using qRT-PCR, how the expression levels varied among the individual genotypes from Population 1 (Figure 5). In this case, the value of P was calculated against the average expression level across genotypes and the validation rate was around 58%. Additional file 3 lists all the values of P for the validated genes.

**Figure 6:**
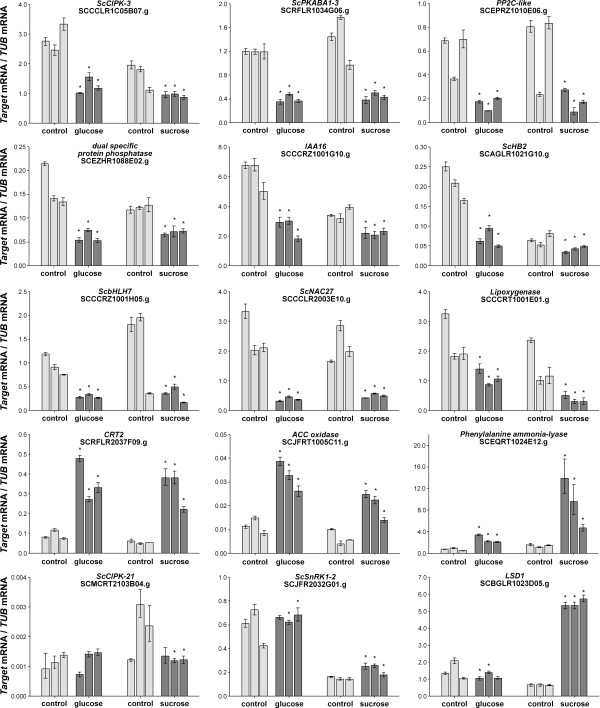
**Quantitative PCR (qRT-PCR) analysis of sucrose and glucose responsive genes**. The y axis refers to the relative expression ratio between target mRNA versus the reference mRNA (tubulin SCCCRZ1002H03.g) for 3 different experiments in sugarcane thirteen-old day seedlings treated with 3% glucose and 3% sucrose for 4 h. R1, R2 and R3 refers to three control and three sucrose and glucose independent treatments. Error bars were calculated as described previously [31]. The transcript levels for the reference genes were verified to not vary in response to the treatments. The values of P correspond to the probability Pr (Treated>Control) and Pr (Treated<Control) for up- and down-regulated genes, respectively. The SAS was considered differentially expressed when P ≥ 0.95.

In order to unravel signaling aspects of sucrose accumulation, we asked whether genes differentially expressed in contrasting Brix genotypes or in mature-versus-immature internodes could represent direct sucrose- and/or glucose-regulated genes and, therefore, be part of the sucrose- and glucose-response pathways. To this end, sugarcane seedlings were treated with 3% sucrose or 3% glucose for 4 h and the expression of thirty-four genes was analyzed by qRT-PCR. The expression of thirty of these genes was affected by sucrose, of which six were also found to be regulated by 3% manitol (osmotic control) and thus, were not considered as true sucrose-responsive genes (see Additional file 3). Figure 6 shows the expression pattern of fifteen of these genes. Among the twenty-four sucrose-regulated genes, nineteen were also found to respond to glucose, indicating a significant overlap between these two signaling pathways (see Additional file 3 and Figure 6). This is not unexpected since sucrose can be readily converted to glucose and sucrose-specific responsive pathways have been identified previously. The five genes, identified here as genuine sucrose-regulated genes, include three SNF1-like kinases, a pathogen-response related protein and a multidrug resistance ABC transporter (see Additional file 3). A weak overlap with ABA signaling was detected, since only three sucrose/glucose-regulated genes were also modulated by ABA (Table 1). Finally, we noticed that thirteen of the twenty-four genes exhibited opposite regulatory responses in high Brix genotypes and/or mature internodes as compared to the short-term sugar-induced regulation in seedlings (data not shown). Together, these data establish the existence of a correlation between high sucrose content and early sucrose and/or glucose-responsive genes, some of which may be relays of signal transduction pathways triggered by these sugars.

In addition, we sought to obtain some insight into the extent to which the short term sucrose and/or glucose regulatory cascade is conserved between sugarcane, a monocot and *Arabidopsis thaliana *(Arabidopsis), a model eudicot organism. Therefore, we compared the data obtained in this study on sugarcane seedlings with results described for *Arabidopsis *seedlings under similar experimental conditions (3% glucose [30] or 0,5% sucrose [34]). Among the twenty-four sugar-regulated sugarcane genes, six of them, along with their eight orthologues in *Arabidopsis *(forming five groups of orthologues) were found to be similarly regulated by glucose and/or sucrose (see Additional file 4 and Additional file 5). The groups of orthologues correspond to SNF1-like kinases (SCRFLR1034G06.g and SCACLR2007G02.g – *At1g78290*), two calreticulin genes (SCRFLR2037F09.g – *At1g56340 *and *At1g09210*), an auxin/IAA transcription factor gene (SCCCRZ1001G10.g – *At3g04730*), a defense and cell wall-related gene encoding a phenyl ammonia-lyase (SCEQRT1024E12.g – *At2g37040*, *At3g53260*, *At3g10340*) and a dehydrin gene (SCQGLR1085F11.g – *At3g50980*) (see Additional file 4). Furthermore, two Arabidopsis genes, the CUC1/NAC-type transcription factor (*At3g1550*) and a wound-responsive gene (*At4g10270*) and their closely related respective sugarcane homologues (SCCCLR2003E10.g and SCCCLR2C01F06.g) were found to be similarly regulated by sugars (Table 1).

## Discussion

Sugarcane partitions carbon into sucrose that can accumulate to 0.7 M in culms [35]. This unique characteristic has been exploited and improved by humans through breeding. Studies that shed light on the molecular mechanisms behind this feature include gene expression and signaling studies on sink and source regulation [36], QTL studies for sucrose accumulation [37] and gene expression profiling during internode maturation [10-12,38]. Such studies indicated that genes associated with sucrose metabolism are not abundantly expressed in culm tissues while genes related to synthesis and catalysis of sucrose are turned off during internode maturation. Genes involved in cellulose synthesis, cell wall metabolism and lignification are also regulated during this process. The activity of genes associated with internode development was evaluated in genotypes of *S. robustum *(which does not accumulate sucrose to high levels), *S. officinarum *and in a hybrid [39]. Mature internodes of all three genotypes showed decreased expression of cell wall metabolism-associated genes and increased expression of genes related to sucrose metabolism. While the general conclusion of these studies does not appear to be in agreement, it is important to note that the genotypes, environment and age of plants used were different and that a larger sampling may be necessary to define gene profiles in sugarcane.

In this work, we evaluated mature and immature internodes of thirty genotypes using cDNA microrrays and qRT-PCR. Genes associated with sucrose content were defined through the analysis of segregating populations selected for one or three generations [40]. Internodes 1, 5 and 9 (In1, 5 and 9) were collected from plants grown in the field. Among the genes found to be differentially expressed were those related to hormone signaling (auxin, ethylene, jasmonates), stress responses (drought, cold, oxidative), cell wall metabolism, calcium metabolism, protein kinases, protein phosphatases and transcription factors. We compared high Brix plants against low Brix plants by hybridizing pairwise the In1, In5 and In9 tissues directly (HB vs LB hybridizations) or by hybridizing mature against immature internodes (MI vs II). We validated gene expression by qRT-PCR in pools of clones and many individual genotypes. We also investigated if genes associated with sucrose content were responsive to sucrose or glucose treatments. Many of the sucrose-associated genes that are regulated during development are associated with drought responses or are modulated by ABA or sugars, as discussed below (see Additional file 2 and Table 1).

### Protein kinases and calcium signaling

Protein phosphorylation appears to play a predominant role in sucrose accumulation and culm development. We have previously categorized sugarcane proteins with a PKinase domain using a phylogenetic approach and named sugarcane protein kinases (PKs) according to the groups obtained, similarity to other kinases and additional domains observed [31]. We now add evidence that several of these genes are regulated during culm development.

A total of fifty-four genes corresponding to PKs, protein phosphatases (PPases) or receptor-like kinases (RLKs) were differentially expressed in high Brix plants or during culm maturation (see Additional file 2). *ScMAPK-4 *(SCSBAM1084E01.g) was more highly expressed in high Brix and mature internodes (Table 1). A MAPK kinase was reported to be involved in the regulation of source metabolism by glucose and stress, which is an indication that *ScMAPK-4 *might be important in establishing sink-source relationships in sugarcane [36,41]. The most predominant PK category altered is the SNF1-like kinase family of proteins. In yeast, SNF1 regulates the expression of genes coding for carbohydrate metabolism and other metabolic enzymes [42]. In plants, SNF1-related kinases have been named SnRK1 [43] and comprise three distinct sub-families (SnRK1, SnRK2 and SnRK3). In sugarcane, we have identified members of all three sub-families [31]. Analogous to SNF1, plant SnRK1s also regulate carbon metabolism at the level of gene expression. At least three important biosynthetic enzymes have been identified as biological substrates of SnRK1s: hydroxymethylglutaryl-CoA reductase (HMG-CoA reductase) [44]; sucrose-phosphate synthase [45] and nitrate reductase [46]. It is possible to make a direct parallel between sucrose accumulation and the gene expression levels for an ScSnRK1 (SCJFRZ2032G01.g). *ScSnRK1-2 *and four 14-3-3 proteins of the GF14 type (SCCCLR1022D05.g, SCCCRZ1001D02.g, SCEQRT1031D02.g and SCEQRT1025D06.g) were expressed at lower levels in mature internodes (Table 1). 14-3-3 proteins, together with a SnRK1, phosphorylate and inhibit the enzyme sucrose phosphate synthase (SPS) *in vitro *[45,47]. Our findings suggest that the decrease in the expression of these genes in the mature internodes may allow for increased sucrose accumulation. We also observed that *ScSnRK1-2 *was induced by sucrose treatment, while most of the *ScCIPKs *and *ScPKABA *and *ScOSAPK *genes were repressed (Table 1). This is an interesting finding that may functionally distinguish the pathways triggered by these kinases in response to sucrose and stress.

Members of the SnRK2 and SnRK3 sub-family including two Osmotic Stress-Activated Kinases – OSA-PK (SCEPRZ1009C10.g and SCCCST1004A07.g) and three CBL-interacting Protein Kinases – CIPK (SCJFRZ2032C08.g, SCMCRT2103B04.g and SCJLRZ1023H04.g) were identified as developmentally regulated during culm maturation (Table 1). Most importantly, three CIPKs (SCBFSB1046D04.g, SCMCRT2103B04.g, SCCCLR1C05B07.g) were more highly expressed in high Brix plants. CBL are regulatory subunits similar to calcineurin that bind to and respond to calcium signals [48]. It has been shown that OSA-PKs and CIPKs mediate drought, osmotic, saline and cold stresses in response to ABA and calcium [49]. Among our differentially expressed genes we found nine genes associated with calcium signaling (SCVPLR1049C09.g, SCCCRZ1C01H06.g, SCJLLR1108H07.g, SCEZLB1012F10.g, SCCCAM1001A03.g, SCAGLR1043F02.g, SCCCCL3120G07.g, SCCCRZ1003A03.g, SCRFLR2037F09.g) and a calcium-dependent protein kinase (SCEQRT2099H01.g – *ScCDPK-27*) that also indicates a role for this second messenger in sucrose accumulation in sugarcane (Table 1). Sucrose synthesis control depends on the activity of the sucrose phosphate synthase, which catalyses the synthesis of sucrose 6-phosphate from UDP-glucose and fructose 6-phosphate. Sucrose breakdown depends on the activity of invertase, which breaks down sucrose into glucose and fructose, and on the activity of sucrose synthase, that converts sucrose in fructose and UDP-glucose in the presence of UDP [35]. Several studies have shown that some CDPKs phosphorylate and regulate sucrose synthase [50-53]. Studies on the maize sucrose synthase showed that phosphorylation of this enzyme on the Ser-15 by CDPKs stimulates the sucrose breakdown activity of this enzyme [50,52]. Besides, CDPKs can phosphorylate residue Ser-170 of this enzyme directing it to the degradation pathway via proteosome 26S [52,54]. The decrease in expression of *ScCDPK-27 *in the mature internode correlates well with increased sucrose in this organ. The activity of sucrose synthase modulates the source-drain relationship [55,56], which eventually determines sucrose content in sugarcane internodes. Additionally, some CDPKs can phosphorylate and inactivate the enzyme sucrose phosphate synthase [57,58], which might contribute to lower sucrose in culms when this enzyme is expressed in high levels, such as seen in low Brix genotypes. Since sucrose biosynthesis is a process regulated by calcium, CDPKs and SnRKs, the genes differentially expressed observed in the high Brix genotypes may all contribute and act as critical control points in sucrose accumulation in this grass.

### Drought signaling

We found a prevalence of gene families regulated by ABA, drought and other stresses among the genes associated with sucrose content [33]. Sixty-nine genes associated with sucrose content were identified to be regulated in response to drought and eleven to ABA (see Additional file 2). This is a strong indication that some of the pathways associated with sucrose content and culm development may overlap with stress signaling pathways. A similar finding was described by Casu and colleagues that found many stress-related genes expressed in culms [11]. Overall, around 32% of the genes previously found to be responsive to drought are associated with sucrose content (Figure 1). It is generally known that sugarcane plants maturation is favoured by the exposure to a period of low water precipitation. It is possible that increased sucrose content is dependent on a drought season and that plants converge the drought and sucrose signaling pathways to sustain growth even during the stress season. Sugars that transduce stress signals or act as osmoprotectants, like fructose, raffinose and trehalose [20] could be central players during this process. A PP from the PP2C family (SCEPRZ1010E06.g) similar to a PPase that transduces the ABA signal was identified as associated with Brix, drought and ABA (Table 1). This PPase was less expressed in high Brix, reduced in the mature internodes and induced by drought and ABA. A similarity search showed that this *PP2C *is very similar to *ABI1 *and *ABI2 *from *Arabidopsis thaliana*. *PP2C*s that are part of the ABA signaling pathway, such as *ABI1*, *ABI2*, *AtPP2CA *and *AtP2CHA*, have their transcript levels increased by this phytohormone [59,60]. Among the processes regulated by ABI1 and ABI2 is stomatal closure, which is certainly one of the first protective measures during drought signaling. Moreover, some of the ABA biosynthesis enzymes are induced by drought and osmotic stress [61]. The fact that sugarcane genes associated with ABA and drought signaling are differentially regulated in plants with distinct sucrose accumulation capacities is an indicative that the role of ABA is well worth being further verified.

Drought responses vary depending on the duration and intensity of the stimulus and comprehend protective alterations and long term growth changes [13]. Many genes responsive to drought have been catalogued [14-18]. The drought stimulus lead to transient calcium fluxes, the activation of calcium sensors, the accumulation of reactive oxygen species, the activation of the MAPK pathway and the induction of several transcription factors including DREB2A, DREB2B [62] and NAC [63]. We have found, among the genes associated with sucrose content, many components of the gene families mentioned above. A MAPK was more expressed in high Brix and in mature internodes and repressed by ABA (SCSBAM1084E01.g), a *DREB *was induced during culm maturation (SCCCLR1001D10.g) (Table 1) and over forty stress responsive genes were identified (Additional file 2). A *DEHYDRIN TYPE 1 *(SCQGLR1085F11.g) regulated by the DREB signaling cascade [64] was dramatically repressed in mature internodes of high and low Brix plants and induced after 72 h and 120 h drought treatment (Table 1). A barley dehydrin gene, *DHN1*, was highly expressed in cells cultured at 25°C and 2°C in media containing high sucrose but our data indicated the dehydrin expression may not be a consequence to high sucrose since immature internodes do not have high levels of this sugar [65]. Overexpression of *DREB2A *in *Arabidopsis thaliana *led to the generation of transgenic plants more tolerant to drought [66,67]. It is possible that some of the genotypes may indeed be more resistant to drought and thus able to grow and accumulate more sucrose, but additional experiments are necessary to verify this hypothesis.

### Transcription factors and hormone signaling

We have recently integrated and evaluated the SUCEST and PlantGDB  EST databases for putative Transcription Factors and identified 2,406 candidate TFs. These were classified into families and can be found at [68]. We found twenty-one transcription factors (TFs) regulated during culm development (Table 1). The great majority was more expressed in the immature internodes, including two members of the Homeobox Knotted1-homeodomain (SCAGLR1021G10.g and SCRLAM1010D08.g), which have been shown to be involved in developmental processes in maize [69]. Developmentally regulated genes include a homolog (SCBFAD1046D01.g) to anthocyanin regulatory R-S protein containing a helix-loop-helix (HLH) domain, that controls tissue-specific synthesis of anthocyanin pigments [70]. Nine transcription factors were identified as differentially expressed when high Brix and low Brix genotypes were compared including an *ARF6 *(*AUXIN RESPONSE FACTOR6a*) (SCEZLB1010E10.g), a *NAM *(*NO APICAL MERISTEM*) (SCCCLR2003E10.g) and an *EIL *(*ETHYLENE INSENSITIVE3-LIKE*) (SCCCRZ1004H12.g) (Table 1). The *NAM *transcript was less abundant in both populations analysed, negatively regulated by sucrose and glucose treatment and induced by drought (Table 1 and Figure 4). NAM transcription factors in *Oryza sativa *have been described as important regulators of drought tolerance [63]. This may indicate a connection between these signaling pathways [71], possible co-regulation associated with sucrose content and cross-talks or signaling overlaps between sugar sensing, sugar mobilization and drought responses.

Among the genes more expressed in the immature internodes, we also found several genes similar to auxin, ethylene and giberellin-responsive TFs (Table 1). We found a second TF related to *ARF6 *(SCCCLR1024F10.g) and four *AUXIN RESPONSE PROTEINS *(SCCCRZ1001G10.g, SCVPLR2005H03.g, SCJFRZ2009F04.g, SCJLLR1054C09.g) more expressed in immature internodes. Signaling by auxins during culm development was also pointed out by the altered expression of two nitrilase genes (SCEQRT1028H06.g and SCRFLR1012D12.g) with a putative role in this hormone's biosynthesis, an auxin efflux carrier (SCCCAM2004G02.g) and a *AUXIN REPRESSED PROTEIN *(SCCCLR2002F08.g) which is up-regulated in mature internodes. One of the nitrilases (SCEQRT1028H06.g) was induced after 72h of drought [31] and its expression level was confirmed by qRT-PCR (Figure 4). Another drought-induced nitrilase (SCCCCL6002B05.g) was found more expressed in high Brix plants. This gene is highly similar to the maize *ZmNIT2 *gene, which converts indole-3-acetonitrile to indole-3-acetic acid [72]. Overall, differential expression of auxin signaling genes during internode development and/or association with sucrose content was observed in twenty different biological samples.

Ethylene was observed as a putative modulator of this process (Table 1). One *EIL *was found less expressed in high Brix plants (SCCCRZ1004H12.g) and two were less expressed in mature internodes (SCBGFL4052C11.g and SCCCRZ1004H12.g). Besides, one ACC oxidase (SCVPLR2012A10.g) was found to be more expressed in high Brix plants and less expressed in mature internodes.

Jasmonic Acid biosynthesis also seems to have a relevant role in culm development since several enzymes envolved in methyl jasmonate biosynthesis were found to be more expressed in immature internodes, two lipoxygenases (SCCCRT1001E01.g and SCJFRT1007H07.g) and an Omega-6 fatty acid desaturase (SCCCLR1C03G01.g) (Table 1).

### Cell wall biosynthesis

There were several genes with a putative function in cell wall metabolism that were among the differentially expressed genes, such as the expansins SCQGRT1040G03.g and SCCCLR2C02A05.g (Table 1). Similar genes were found to be expressed in two-day-old rice seedlings, a stage where rapid cell elongation occurs accompanied by cell division [73]. The authors believe EXP activity may be required for cell expansion. Expansins may act in the relaxation of the cell wall, possibly by breaking the bonds between cellulose microfibrils and matrix polysaccharides [74,75] allowing for cell expansion. Our data indicates that, in sugarcane, a gene similar to *EXPA23 *(SCQGRT1040G03.g) is more expressed in 7-month old high Brix plants as compared to low-Brix plants, and the *EXPA11 *(SCCCLR2C02A05.g) in turn, is more expressed in low Brix plants (after 11 months of planting) (Table 1). As mentioned above, we have evidence that auxin signaling is highly active in immature internodes. Auxin signaling is associated with plant cell expansion [76], which may be an additional evidence that the high Brix plants selected have cell expansion alterations that might confer higher sucrose accumulation capacity. These observations are corroborated by the identification of a *XYLOGLUCAN ENDO-B-1,4 GLUCANASE *(*XTH*) (SCBFLR1039B05.g) that is more expressed in immature internodes (Table 1). XTHs can hydrolyse xyloglucans, major components of plant cell walls, and transglycosylate residues into growing xyloglucan chains, that may be important during tissue expansion [77]. Our data is in agreement with previous findings from Casu and colleagues [12] that identified five *XTHs *and four β-expansins less expressed in mature internodes, as well as two caffeoyl-CoA O-methyltransferases induced in mature internodes.

We found five genes of the lignin biosynthesis pathway associated with sucrose content (Table 1). The first step in lignin biosynthesis in plants is the deamination of L-phenylalanine by Phenylalanine Ammonia-Lyase (PAL) to cinnamic Acid. PAL is the first enzyme of the phenylpropanoid pathway [78]. It converts L-phenylalanine into *trans*-cinnamic acid (*t*-CA), which is further transformed in plants into many phenylpropanoid compounds, such as lignins, antioxidants, anthocyanins and flavonoid nodulation factors. We found three *PAL *genes associated with sucrose content and more expressed in immature internodes (SCCCLR1048D07.g, SCEQRT1024E12.g and SCSGAM1094D05.g). SCEQRT1024E12.g was induced after ABA treatment and repressed after 72 and 120 h drought stress. The second step in lignin biosynthesis is catalyzed by a cinnamate 4-hydroxylase (C4H) [79]. We found one SAS less expressed in high Brix similar to a *C4H *(SCSGFL4193B05.g). Down the pathway *p*-Coumaroyl is transformed into Caffeoyl CoA by a *p*-coumaroyl shikimate 3'-hydroxylase (C3H). One SAS similar to a *C3H *(SCACSB1037A07.g) was found less expressed in high Brix plants. We also observed a *FERULATE 5-*HYDROXYLASE (*F5H*) (SCEZHR1087F06.g) and a *CAFFEIC ACID 3-O-METHYLTRANSFERASE *(*COMT*) (SCRFLR1012F12.g) more expressed in mature internodes. *F5H *was less expressed in high Brix while *COMT *was induced (Table 1). While all the above mentioned genes may have a role in cell wall metabolism, it is important to note that *trans*-cinnamic acid can also be converted into salicylic acid and anthocyanins [80] and, until the activity of these enzymes is verified, the data can only indicate a putative alteration in cell wall biosynthesis and modification in the accumulation of sucrose in culms.

Cell wall biosynthesis can reduce sucrose accumulation since carbon fluxes directed to plant growth and cell wall expansion may alter carbon partitioning into sucrose. It is also possible that sucrose accumulation may trigger increased lignification. One of the PAL enzymes was induced by sucrose treatment up to 14-fold indicating that this enzyme is highly responsive to sucrose. An induction of a *COMT *gene has already been described during culm maturation [12] but this is the first report implicating a PAL, C4H, C3H, F5H and COMT in sucrose content. It is possible that some of the genotypes analyzed also differ in biomass content and a continued agronomic evaluation is necessary to assess how gene expression in the selected genotypes is related to other characteristics, such as cell wall composition, growth rates, internode size and width, number of internodes and drought tolerance, for instance. Many parameters besides Brix may differ among the genotypes and have not yet been evaluated. It is possible also that the high Brix genotypes may be more amenable for acid and enzymatic hydrolysis and cellulosic ethanol production. Silencing of lignin biosynthesis genes has been shown to benefit sugar release for lignocellulosic biomass fermentation [81]; it will be thus interesting to test if altered biomass has been selected for during the breeding process. At any rate, the alteration of these cell wall biosynthesis genes in association to Brix content is an interesting indication of a correlation between these processes.

### A relationship between high sugar content and sugar signaling

We showed that a set of genes associated with sucrose content is also early sugar-responsive. Since most of these genes are related to signal transduction (kinases, phosphatases, transcription factors, hormone synthesis) they are likely to constitute upstream components of the sugar regulatory cascade. These findings raise the interesting possibility that sugar signaling may somehow influence sugar accumulation capacity in sugarcane. How these genes may influence sucrose accumulation is an open question. Interestingly, between sucrose/glucose-treated young seedlings and high Brix genotypes, contrasting expression patterns were found for 15 genes, while the remaining 9 genes presented similar regulatory trends (Table 1 and data not shown). This latter set of genes may be related to higher sugar fluxes and/or higher sugar sensitivity in high Brix genotypes. Opposite regulatory patterns between young seedlings and internodes of high Brix genotypes are more difficult to explain, but could reflect differential developmental-dependent controls. A comparative analysis with *Arabidopsis *showed that among the twenty-four sugarcane genes tested, five *Arabidopsis*-sugarcane probable groups of orthologues and two pairs of *Arabidopsis*-sugarcane close homologues (sister clades) were apparently regulated similarly by sugars in seedlings (see Additional file 4). Orthologous genes of the signal transduction-class, such as those encoding transcription factors or kinases with conserved regulatory features, are likely to represent important players in the sugar signal transduction pathways and this can now be tested. Within this framework, it should also be interesting to further analyze the integration/interaction of the *Arabidopsis *CUC1/NAC-type transcription factor (At3g1550), which controls shoot apical meristem formation [82] in the sugar regulatory network.

### Data validation across genotypes

To confirm gene expression and evaluate transcript levels we performed qRT-PCR reactions for forty-two genes. With a probability value higher than 0.95 we observed that 80% of the gene expression data obtained using cDNA microarrays were compatible with the qRT-PCR data.

Validation of developmental regulation was elucidative of differences among high Brix and low Brix populations. *ScCIPK-21 *for instance, a gene more expressed in high Brix and in mature internodes, was found to be much more induced during culm development in the high Brix plants, what may be an indicative that induction of this gene may lead to higher sucrose levels. A category that was consistently more expressed in immature internodes and high Brix and that has been seen to be responsive to drought in other plants is the aquaporin family of proteins. We wanted to verify if genes of this family could be useful expression markers of sucrose content. Five aquaporins, from both the MIP and PIP sub-families (SCCCRZ1002E08.g, SCCCST3001H12.g, SCEQRT2100B02.g, SCCCLR1024C03.g, and SCCCRZ1001F02.g) were regulated during culm development and two of them found to be associated with high Brix (SCCCST3001H12.g and SCEQRT2100B02.g) in population 2 (Table 1). This large family of proteins is primarily involved in the regulation of water movement between cells and cell compartments, although many of them also facilitate the passage of small solutes [83,84]. The accumulation of sucrose in such high concentrations as seen in sugarcane cells certainly represents an osmotic challenge that demands efficient control of solute compartmentalization and water transport out of the vacuoles. As key players in the equilibration of water potentials via regulation of membrane permeability, aquaporins may have a fundamental role in the process of sugar storage in sugarcane vacuoles. It has been observed in *Arabidopsis *that loss of the aquaporin TIP1.1 severely affects carbohydrate metabolism and transport [85], and the authors postulate that this aquaporin could be involved in a vesicle-based routing of carbohydrates towards the central vacuole. In our study, expression of one aquaporin correlated to lower sucrose content in both populations analyzed. Since differential expression was determined in pools of seven or eight individuals we decided to verify how many of the genotypes in those pools presented the observed expression patterns. Expression data was obtained using qRT-PCR for twelve of the sixteen extreme individuals of Population 1 (Figure 5). We calculated the average expression levels across all twelve genotypes for the Aquaporin gene (SCCCRZ1002E08.g) and observed that the gene was less expressed in all high Brix genotypes and more expressed in half of the low Brix genotypes. This is a strong indicative that low expression of this gene has been segregated and selected by the breeding process and is strongly associated with high sucrose content. To verify if this would be the case for other differentially expressed genes we evaluated the expression profiles of eleven genes in the twelve extreme genotypes of Population 1. For some genes, such as the HLH TF (SCCCRZ1001H05.g), expression was consistently lower than average in all low Brix genotypes, but found to be higher in only two of the high Brix genotypes (Figure 5). In this case, the two genotypes had almost 10,000 fold increased expression for the genes, which may account for the differential expression observed in the pool used for cDNA microarray analysis. This is an extreme case and, in general, most of the data shows around 50% of validation in the individual genotype validations.

## Conclusion

Gene expression analysis of sugarcane populations contrasting for sucrose content indicated a possible overlap of sugar, drought and cell wall metabolism processes and suggested signaling and transcriptional regulators that might be useful as molecular markers in breeding programs or as primary targets in a sugarcane improvement program based on transgenic plants. This work is a first survey on gene expression related to sucrose content and some similarities point to conservation between monocot and dicot sucrose responses. This observation can help to point the important players in the sugar signal transduction pathways. Due to the diversity of roles described for the identified genes, additional experiments will be necessary to elucidate their possible roles in the sugarcane sucrose accumulation process. Our group is currently generating transgenic plants with modified expression levels for these genes to confirm the hypothesis raised for their function.

## Methods

### Biological samples

Population 1 was derived from two intra-specific polycrosses, one among 21 *Saccharum officinarum *genotypes (Caiana Fita, IK76108, Lahaina, MZ151, MZ151 roxa, Sabura, Salangor, Sinimbu, NG213, Fiji 47, Hinahina 18, Manjri Red, Muntok Java, NG77142, Soff 8268, SS601, Sylva, NG2880, Vae Vae Ula, IJ76315 and IN8425) and the other combining 13 *Saccharum spontaneum *genotypes (IN8458, IN8488, Krakatau, SES 147b, US56158, US7440, US851008, UM721, UM691, SES 194, IK7686, US56193 and US571723). The individuals of these polycrosses were crossed amongst themselves and for each generation, 500 individuals were sampled for soluble solids (Brix degree). The extreme segregants of the F3 hybrid individuals were planted in a field in single rows of 5 m using standard sugarcane cultivation practices. Tissue samples were collected in March of the following year, when plants were 10 months old. The Brix degree content of the 4^th^–5^th ^internodes of each sugarcane stalk was measured with a portable refractometer (N1 model, ATAGO, Japan). Additional file 1 lists Brix measurements for the extreme individuals of both populations [33] and the corresponding sucrose concentration. The average Brix value for high sugar individuals was 18.10 +/- 1.44 and for low sugar individuals was 6.70 +/- 0.96 for Population 1. Sucrose content was 9.2% in high Brix individuals and 1.1% in low Brix individuals for this population. Sugars were determined as described [86,87].

Population 2 was derived from a cross between two commercial varieties (SP80-180 × SP80-4966). Five hundred sugarcane F1 plants were field-grown. Seven plants with extreme Brix values were selected. Population 2 was less contrasting than Population 1, with an average high Brix of 18.47 +/- 1.41 and average low Brix of 13.65 +/- 1.27.

Sucrose accumulating tissues (sink tissues, herein internodes) were collected from field grown plants. We have previously determined using Pair-wise Pearson correlation calculations a high correlation of gene expression between individuals collected at the same time or within a short interval of time (0.84 to 0.88), and a lower correlation between individuals collected in different years (0.61 to 0.64) [88]. Mature (In9), intermediate (In5) and immature internodes (In1) were then collected from four selected plants of each genotype at 7, 10 and 11 months after planting. Tissue collected from the four plants was pooled, therefore each biological sample corresponds to a certain tissue derived from four plants, and the gene expression data reflects the average expression of the pooled plant tissue. A total of 132 biological samples were selected for gene expression studies from both populations. RNA was extracted from tissues of individuals or pools of eight individuals as described [31].

The cultivar SP90-1638 (Internal Technical Report, CTC, 2002), sensitive to drought, was used for the water deprivation experiments. The experiments were previously described [31]. Briefly, plants were transferred to pots containing moist sand under greenhouse conditions. Regular watering was maintained for 90 days, being suppressed after this period for the experimental group. Aerial parts of six plants for each experimental point were collected 24, 72 and 120 h after the onset of drought for the control and experimental groups.

For ABA treatment, plants derived from shoot apex of 2-month-old sugarcane plants were *in vitro *cultivated for approximately three months in a growth chamber as described [31]. ABA (Sigma Chem. Co) was added to the culture medium to a final concentration of a 100 μmol.L ^-1 ^whereas control plants were treated with distilled water. Leaves were collected after 0.5h, 1h, 6h and 12 h of exposure to ABA. Six plantlets were sampled for each time point.

For sucrose treatment, seeds obtained from a crossing between SP891046 and IAC912195 varieties were imbibed in water, incubated for 10 min at 52°C to open the panicle and sterilized by a 5-min treatment in 70% ethanol followed by 20-min in 2.5% sodium hypoclorite. Seeds were then washed 5 times in sterile water and then transferred to a Musharige and Skoog half-strength solid growing media [89] containing 0.5% of sucrose. Plates were incubated in continuous light for 12 days at 28°C. Subsequently, _seedlings were transferred to liquid MS/2 growth medium without any sugar and further grown for 24 h under weak agitation (60 rpm) and constant light before being treated with 3% sucrose or 3% glucose or 3% mannitol (stock solution of 30% in water) or just with water as control for 4 h. RNA was extracted using Concert^® ^(Invitrogen, USA) according to the manufacturer's recommendations.

### Gene expression data

cDNA microarray experiments were conducted and data extracted as described previously [31]. SUCEST SAS consensus sequences can be found at . The corresponding Sugarcane Gene Index contigs  can be searched at  and downloaded at  and . The designed microarray contains 1830 genes which yielded 1545 good-quality PCR fragments. Reverse transcription, labeling and hybridizations were done using the reagents provided with the CyScribe Post-Labeling kit (GE Healthcare) or SuperScript™ Plus Indirect cDNA Labeling System (Invitrogen, USA). The microarrays were scanned according to the manufacturer's instructions using the Generation III System (Molecular Dynamics). Hybridizations were carried out as described [33].

Two technical replicates were obtained for each microarray experiment. Data were collected using the ArrayVision (Imaging Research Inc.) software. The fluorescence ratios were normalized in the MxS space, where M is the base 2 logarithm of the intensities ratio and S is the base 2 logarithm of the average intensity of each spot. The M values were normalized to account for systematic errors using the LOWESS fitting. The identification of differentially expressed genes was performed using a local implementation of the Outliers Search method [31]. The SAS presenting more than 70% of its replicates outside fold-change cut-off curves were defined as differentially expressed. Raw data can be found at .

### Validation of microarray results by real-time PCR (qRT-PCR)

Real-time PCR reactions were done essentially as described [31]. The ratio between the relative amounts of the target gene and the endogenous control gene in the qRT-PCR reactions was determined based on the Ct method [90] with modifications. The normalized expression level was calculated as L = 2^-ΔCt ^and ΔCT = C_T, target _- C_T, reference_. A polyubiquitin (PUB) gene (SCCCST2001G02.g) was used as an endogenous reference in the qRT-PCR reactions of high Brix and low Brix samples after verification that its mRNA levels were similar in the populations and individual tissues. This PUB gene was also used for the sucrose-responsive gene expression validation. Drought samples were normalized using a GAPDH (CA254672.1 [91]), PUB (SCCCST2001G02.g) or Ubiquitin (SCCCLR1048F12.g) genes and ABA samples using a UBE2 (*ubiquitin conjugating enzyme E2*) (SCBGLR1002D06.g) or PUB gene (SCCCST2001G02.g).

To access the statistical significance of expression ratios, we assumed a log-normal model and calculated the probability P = Pr(sample>reference) and P = Pr(sample<reference) for up- and down-regulated genes, respectively. The expression profile was considered validated when P ≥ 0.95. For validation of gene expression differences among all different genotypes the probability value P of being greater or smaller than the average expression across all individuals was calculated depending on whether the condition was respectively up- or down-regulated according to the microarray data.

### Comparative sequence analysis

Comparative analysis of sugarcane sugar-responsive genes was done by constructing phylogenetic trees containing the corresponding most similar plant sequences. A tblastx search [92] with the sugar-regulated SAS against a green plants protein data set including 365,187 proteins sequences obtained from several genomes (*Arabidopsis thaliana*, version 7.0 – ; *Populus trichocarpa*, version 1.1 – ; *Glycine max*, version 0.1 – ; *Oryza sativa*, version 5.0 – ; *Sorghum bicolor*, version 1.4 – ; *Selaginella moellendorffii*, version 1.0 – ; *Physcomitrella patens patens*, version 1.1 – ; *Volvox carteri*, version 1.0 – ; Chlamydomonas reinhardtii, version 3.0 – ; *Ostreococcus lucimarinus*, version 2.0 – ; *Ostreococcus tauri*, version 2.0 – ; *Micromonas pusilla CCMP1545*, version 2.0 – ; *Micromonas strain RCC299*, version 2.0 – ) was performed. For each SAS, the first 40 best matches, or all matches obtained if this number were lower than 40, were selected for further analysis. The conserved domains found among the 40 selected sequences were aligned using ClustalW [93] to produce ungapped alignments. The phylogenetic relationship of these aligned sequences was then constructed using the Neighbor-Joining method [94] using p-distance. All analysis were conducted in MEGA4 software [95]. This process allowed identifying the most probable orthologues sequences of the SAS. The Arabidopsis orthologues and the Arabidopsis more closely related homologues (usually one sister clade which may include at least two Arabidopsis sequences) were compared with the set of Arabidopsis genes regulated by glucose [30] and/or sucrose [34] using VennMaster 0.37.3 .

## Authors' contributions

FSPT, FRR, AJW, CGL, MDLC and DB conducted cDNA microarray and qRT-PCR experiments. LEVDB conducted the phylogenetic analysis. ECU was responsible for sugarcane cultivation and germplasm sample collection. MYNJ, RZNV and RV were responsible for bioinformatic analysis and database development. MV, MM and GMS are group leaders, designed the experiments, analysed the data and had intelectual input in all activities listed above. All authors contributed to datamining, read the manuscript and approved it.

## Note

**Additional files **can be found at 

**Raw data **has been uploaded to GEO Database (Series GSE14732)

## Supplementary Material

Additional file 1 **Brix degree and sugar content of populations**. Brix degree, sucrose, glucose and fructose were determined from 10-month old plants of Population 1 and 11-month old plants of Population 2. The measurements were made from juice extracted from the 9^th ^internode. Brix measurements of these populations have been previously described [33] Click here for file

Additional file 2 **SAS showing differential expression when high and low Brix plants were compared or when mature and immature internodes were compared using cDNA microarrays**. The table also shows differential expression of the same SAS as seen in [31] for plants submitted to drought and ABA treatment. The table lists a SAS whose expression was enriched or decreased as determined by the Outliers Search Method in two technical replicates for each biological sample. The expression ratio for each technical replicate is in brackets. Click here for file

Additional file 3 **P value of qRT-PCR**. Genes associated with sucrose content, drought, ABA and sugars were validated by qRT-PCR. The tables indicate all the genes evaluated and the values of P for differential expression. Click here for file

Additional file 4 **Sugarcane and Arabidopsis orthologues similarly regulated by sucrose and glucose**. Orthologies between Sugarcane and Arabidopsis were assigned using the Neighbor-Joining method [94]. The Arabidopsis orthologues were compared with the set of Arabidopsis genes regulated by glucose [30] and/or sucrose [34]. Click here for file

Additional file 5 **Inferred phylogenetic relationships among tblastx hits using the sugarcane SAS as queries**. The amino acid alignments were performed with ClustalX. The distances were obtained by p-distance and topography inferred with Neighbor-Joining (NJ) using only the aligned blocks (complete deletion). Analysis were conducted in MEGA4. The continuous blocks show regulation by sucrose and the pointed blocks show regulation by glucose (in both cases red for induction and green for repression). **A **– SCRFLR2037F09.g (Calreticulin 2); **B **– SCEQRT1024E12.g (Phenylalanine ammonia-lyase); **C **– SCCCRZ1001G10.g (IAA16); **D **– SCACLR2007G02.g and SCRFLR1034G06.g (canePKABA1-1 and canePKABA1-3); **E **– SCQGLR1085F11.g (Dehydrin). The sequences names correspond to those present in the protein data sets showed in Material & Methods: **AT **– *Arabidopsis thaliana*; **Gm **– *Glycine max *(soybean); **jgi|Poptr1 **– *Populus trichocarpa*; **LOC Os **– *Oryza sativa *(rice); **Sb **– *Sorghum bicolor *(sorghum); **jgi|Selmo1 **– *Selaginella moellendorffii*; **jgi|Phypa1_1 **– *Physcomitrella patens patens*; **jgi|MicpuC2 **– *Micromonas pusilla CCMP1545*, **jgi|MicpuN2 **– *Micromonas strain RCC299*; **jgi|Volca1 **– *Volvox carteri*; **jgi|Chlre3 **– *Chlamydomonas reinhardtii*. Click here for file
