# Supplementary material for: Sugarcane genes associated with sucrose content
Source: BMC Genomics. 2009 Mar 21;10:120. doi: 10.1186/1471-2164-10-120 (PMC2666766; doi:10.1186/1471-2164-10-120)
Supplement: Additional file 3 — P value of qRT-PCR. Genes associated with sucrose content, drought, ABA and sugars were validated by qRT-PCR. The tables indicate all the genes evaluated and the values of P for differential expression. [file 1471-2164-10-120-S3.doc]

**Additional file 4**

P values of qRT-PCR of High Brix vs Low Brix samples (pooled genotypes)

| **SAS** | **Category** | **sub category 1** | **sub category 2** | **p Value** | | | | | |
| --- | --- | --- | --- | --- | --- | --- | --- | --- | --- |
|  |  |  |  | **Up in HB pool** | | | | | |
|  |  |  |  | **Progenie 1** | | | **Progenie 2** | | |
|  |  |  |  | **In 1** | **In 5** | **In 9** | **In 1** | **In 5** | **In 9** |
| SCCCAM1001A03.g | Calcium | Calmodulin-binding proteins | Multidrug resistant-like |  |  | 1.00 |  |  |  |
| SCSBHR1050B11.g | development | putative senescence-associated protein | . |  | 0.98 |  |  | 0.81 |  |
| SCJFRT1005C11.g | hormone biosynthesis | Ethylene | ACC oxidase |  |  |  | 1.00 |  |  |
| SCEQRT1024E12.g | hormone biosynthesis | Salicylic Acid | . |  |  |  | 0.96 |  |  |
| SCEZRZ3098G10.g | Pathogenicity | Protease inhibitors | Thaumatin |  |  | 1.00 |  |  |  |
| SCEQRT1028C03.g | Pathogenicity | R-genes transduction | PR |  |  |  | 1.00 |  | 1.00 |
| SCBFSB1046D04.g | protein kinases | SNF-like kinases | ScCIPK-16 |  |  | 0.89 |  |  |  |
| SCMCRT2103B04.g | protein kinases | SNF-like kinases | ScCIPK-21 |  |  |  |  | 1.00 | 0.00 |
| SCCCLR1C05B07.g | protein kinases | SNF-like kinases | ScCIPK-3 |  |  |  |  | 1.00 |  |
| SCEZHR1088E02.g | protein phosphatases | Tyrosine Phosphatases | Dual Specificity Protein Phosphatases (DSPP) |  |  |  | 0.99 | 1.00 | 0.00 |
| SCJFRZ2012F04.g | Receptors | Receptor Ser/Thr kinase | ScRLK-DIV3 |  |  | 0.99 |  |  |  |
| SCEQRT2100B02.g | Stress | Drought and cold response | putative aquaporin (tonoplast intrinsic protein) |  |  |  |  |  | 1.00 |
| SCQGHR1012B09.g | Stress | Probable cytochrome P450 monooxygenase | . |  | 0.00 |  |  |  |  |
| SCCCLR2C01F06.g | Stress | Wound-induced | . |  |  |  | 1.00 |  | 1.00 |
| SCCCRZ1001H05.g | transcription factor | HLH (helix-loop-helix) | ScbHLH7 | 0.91 |  |  |  | 0.99 |  |
|  |  |  |  |  |  |  |  |  |  |
|  |  |  |  | **Down in HB pool** | | | | | |
|  |  |  |  | **Progenie 1** | | | **Progenie 2** | | |
|  |  |  |  | **In 1** | **In 5** | **In 9** | **In 1** | **In 5** | **In 9** |
| SCRFLR2037F09.g | Calcium | Calreticulin | . | 1.00 |  |  |  |  |  |
| SCCCRT1001E01.g | hormone biosynthesis | Jasmonic Acid | Lipoxygenase |  |  |  | 1.00 |  |  |
| SCRUFL1112F04.b | Others | RNA stability | UDP-GlcNAc |  |  | 1.00 |  |  |  |
| SCEPRZ1010E06.g | protein phosphatases | Serine/Threonine - PPM Family | PP2C-like |  |  |  | 1.00 |  | 1.00 |
| SCCCLR2003E10.g | Transcription | NAM | ScNAC27 |  |  | 1.00 |  | 1.00 |  |

P values of qRT-PCR of internodes development samples.

| **SAS** | Category | sub category 1 | sub category 2 | **p Value** | | | | | |
| --- | --- | --- | --- | --- | --- | --- | --- | --- | --- |
|  |  |  |  | **Up in mature internodes** | | | | | |
|  |  |  |  | **HB pool** | | | **LB pool** | | |
| SCMCRT2103B04.g | protein kinases | SNF-like kinases | ScCIPK-21 | 1.00 | | | 1.00 | | |
| SCCCCL3120C09.g | receptors | Receptor Ser/Thr kinase | ScRLK with LysM-1 | 0.01 | | | 0.39 | | |
|  |  |  |  |  |  |  |  |  |  |
|  |  |  |  | **Down in mature internodes** | | | | | |
|  |  |  |  | **HB pool** | | | **LB pool** | | |
| SCCCRZ1001D02.g | adapters | 14-3-3 proteins | . | 1.00 | | | 1.00 | | |
| SCBFLR1039B05.g | carbohydrate Metabolism | Xyloglucan endotransglycosylase | . | 1.00 | | | 1.00 | | |
| SCEQRT1028H06.g | hormone biosynthesis | Auxin | Nitrilase | 0.00 | | | 0.00 | | |
| SCJFRT1005C11.g | hormone biosynthesis | Ethylene | ACC oxidase | 1.00 | | | 1.00 | | |
| SCCCRT1001E01.g | hormone biosynthesis | Jasmonic Acid | Lipoxygenase | 1.00 | | | 1.00 | | |
| SCBFST3136A06.g | No matches | . | . | 0.04 | | | 0.31 | | |
| SCBGLR1023D05.g | pathogenicity | R-genes transduction | LSD1 | 1.00 | | | 1.00 | | |
| SCEPRZ1009C10.g | protein kinases | SNF-like kinases | cane osmotic stress-activated protein kinase-1 | 1.00 | | | 1.00 | | |
| SCCCRT2001H11.g | Small GTPases | Arf | . | 1.00 | | | 1.00 | | |
| SCCCRZ1002E08.g | Stress | drought and cold response | putative aquaporin | 1.00 | | | 1.00 | | |
| SCQGLR1085F11.g | Stress | Drought-induced | DHN | 1.00 | | | 1.00 | | |
| SCCCLR1024E11.g | Stress | Superoxide dismutases | Cu/Zn | 0.33 | | | 0.00 | | |
| SCCCRZ1001G10.g | transcription | Aux/IAA | IAA16 | 1.00 | | | 1.00 | | |
| SCSGSB1009D11.g | Unknown protein | . | . | 1.00 | | | 1.00 | | |

P values of qRT-PCR of genotypes from progeny 1 (individual genotypes).

| **SAS** | **category** | **sub category 1** | **sub category 2** | **Inter**  **node** | **p Value** | | | | | | | | | | | |
| --- | --- | --- | --- | --- | --- | --- | --- | --- | --- | --- | --- | --- | --- | --- | --- | --- |
|  |  |  |  |  | **Up in HB genotypes** | | | | | | **Down in LB genotypes** | | | | | |
|  |  |  |  |  | **CTC98-241** | **CTC98-242** | **CTC98-243** | **CTC98-244** | **CTC98-246** | **CTC98-253** | **CTC98-261** | **CTC98-262** | **CTC98-265** | **CTC98-272** | **CTC98-277** | **CTC98-279** |
| SCCCAM1001A03.g | calcium | Calmodulin-binding proteins | Multidrug resistant-like | 9 | 1.00 | 1.00 | 1.00 | 0.88 | 0.00 | 1.00 | 0.03 | 1.00 | 0.00 | 0.00 | 1.00 | 1.00 |
| SCSBHR1050B11.g | development | putative senescence-associated protein | . | 5 | 0.42 | 0.04 | 1.00 | 0.00 | 1.00 | 1.00 | 0.00 | 1.00 | 0.00 | 0.00 | 1.00 | 0.99 |
| SCEZRZ3098G10.g | pathogenicity | Protease inhibitors | thaumatin | 9 | 1.00 | 1.00 | 1.00 | 0.77 | 1.00 | 1.00 | 1.00 | 1.00 | 0.00 | 1.00 | 1.00 | 1.00 |
| SCBFSB1046D04.g | Protein kinases | SNF-like kinases | ScCIPK-16 | 9 | 1.00 | 0.01 | 0.01 | 1.00 | 1.00 | 1.00 | 0.00 | 1.00 | 0.00 | 0.52 | 1.00 | 1.00 |
| SCJFRZ2012F04.g | receptors | Receptor Ser/Thr kinase | ScRLK-DIV3 | 9 | 0.00 | 1.00 | 1.00 | 0.30 | 1.00 | 1.00 | 1.00 | 1.00 | 0.00 | 0.00 | 1.00 | 1.00 |
| SCQGHR1012B09.g | Stress | Probable cytochrome P450 monooxygenase | . | 5 | 0.00 | 0.01 | 1.00 | 0.00 | 1.00 | 1.00 | 0.00 | 1.00 | 0.00 | 0.00 | 1.00 | 0.98 |
| SCCCRZ1001H05.g | transcription factor | HLH (helix-loop-helix) | ScbHLH7 | 1 | 1.00 | 1.00 | 0.00 | 0.00 | 0.00 | 0.00 | 1.00 | 1.00 | 1.00 | 1.00 | 1.00 | 1.00 |
|  |  |  |  |  |  |  |  |  |  |  |  |  |  |  |  |  |
|  |  |  |  |  | **Down in HB genotypes** | | | | | | **Up in LB genotypes** | | | | | |
|  |  |  |  |  | **CTC98-241** | **CTC98-242** | **CTC98-243** | **CTC98-244** | **CTC98-246** | **CTC98-253** | **CTC98-261** | **CTC98-262** | **CTC98-265** | **CTC98-272** | **CTC98-277** | **CTC98-279** |
| SCRFLR2037F09.g | calcium | Calreticulin | . | 1 | 1.00 | 1.00 | 1.00 | 1.00 | 0.00 | 1.00 | 1.00 | 1.00 | 1.00 | 1.00 | 0.92 | 1.00 |
| SCRUFL1112F04.b | Other | RNA stability | UDP-GlcNAc | 9 | 0.02 | 0.00 | 0.01 | 0.00 | 0.00 | 0.00 | 1.00 | 0.00 | 1.00 | 0.38 | 0.00 | 0.00 |
| SCCCRZ1002E08.g | Stress | Drought and cold response | putative aquaporin (tonoplast intrinsic protein) | 9 | 0.92 | 1.00 | 1.00 | 0.97 | 0.96 | 0.98 | 0.88 | 1.00 | 1.00 | 1.00 | 0.62 | 0.66 |
| SCCCLR2003E10.g | transcription factor | NAM | ScNAC27 | 9 | 1.00 | 0.00 | 0.00 | 0.00 | 0.00 | 1.00 | 1.00 | 0.00 | 1.00 | 1.00 | 0.00 | 0.00 |

**P values of qRT-PCR of drought stress and ABA treatment samples**

| **SAS** | **Category** | **sub category 1** | **sub category 2** | **p Value** |
| --- | --- | --- | --- | --- |
| **Drought Stress** |  |  |  | **Up in Drought** |
| SCEQRT1028H06.g | hormone biosynthesis | Auxin | Nitrilase | 1.00 |
| SCEZHR1088E02.g | Protein Phosphatases | Tyrosine Phosphatases | Dual Specificity Protein Phosphatases (DSPP) | 1.00 |
| SCEPRZ1010E06.g | Protein Phosphatases | Serine/Threonine - PPM Family | PP2C-like | 1.00 |
| SCQGLR1085F11.g | stress | Drought-induced | . | 1.00 |
|  |  |  |  |  |
| **Drought Stress** |  |  |  | **Down in Drought** |
| SCEQRT1024E12.g | hormone biosynthesis | Salicylic Acid | . | 1.00 |
| SCBFST3136A06.g | No matches | . | . | 1.00 |
|  |  |  |  |  |
| **ABA treatment** |  |  |  | **Up in ABA** |
| SCEQRT1024E12.g | hormone biosynthesis | Salicylic Acid | . | 1.00 |
| SCRUSB1062E12.g | lipid metabolism | Putative triacylglycerol lipase | . | 1.00 |
| SCEPRZ1010E06.g | Protein Phosphatases | Serine/Threonine - PPM Family | PP2C-like | 1.00 |
|  |  |  |  |  |
| **ABA treatment** |  |  |  | **Down in ABA** |
| SCAGLR1043E04.g | Stress | cytochrome P450 | CYP74A | 1.00 |
| SCEQRT1028H06.g | hormone biosynthesis | Auxin | Nitrilase | 0.93 |

**P values of qRT-PCR of sucrose treatment samples.**

| **SAS** | **Category** | **sub category 1** | **sub category 2** | **p Value** | | | | | |
| --- | --- | --- | --- | --- | --- | --- | --- | --- | --- |
| **Up regulated** |  |  |  | **Sucrose** | | | **Glucose** | | |
|  |  |  |  | **R1** | **R2** | **R3** | **R1** | **R2** | **R3** |
| SCCCAM1001A03.g* | calcium metabolism | calmodulin-binding protein | Multidrug resistant (MDR) ABC transporter | 0.99 | 1.00 | 0.78 | - | - | - |
| SCRFLR2037F09.g | calcium metabolism | calreticulin | CRT2 Calreticulin 2 | 1.00 | 1.00 | 1.00 | 1.00 | 1.00 | 1.00 |
| SCJFRT1005C11.g | hormone biosynthesis | ethylene | ACC oxidase | 1.00 | 1.00 | 1.00 | 1.00 | 1.00 | 1.00 |
| SCEQRT1024E12.g | hormone biosynthesis | salicylic acid | Phenylalanine ammonia-lyase | 1.00 | 1.00 | 1.00 | 1.00 | 1.00 | 1.00 |
| SCBGLR1023D05.g* | pathogenicity | R-gene transduction | Zinc finger protein (LSD1) | 1.00 | 1.00 | 1.00 | - | - | - |
| SCJFRZ2032G01.g* | protein kinase | SNF-like kinase | ScSnRK1-2 | 1.00 | 1.00 | 0.97 | - | - | - |
| SCCCRT2001H11.g | small GTPase | ARF (ADP-ribosylation factor) | ARF1 | 0.82 | 1.00 | 1.00 | 1.00 | 1.00 | 1.00 |
| SCCCLR2C01F06.g | stress | wound-induced | wound-responsive family protein | 1.00 | 1.00 | 1.00 | 1.00 | 1.00 | 1.00 |
|  |  |  |  |  |  |  |  |  |  |
| **Down regulated** |  |  |  | **Sucrose** | | | **Glucose** | | |
|  |  |  |  | **R1** | **R2** | **R3** | **R1** | **R2** | **R3** |
| SCJLHR1028C12.g | DNA metabolism | histone | Histone H4 | 1.00 | 1.00 | 1.00 | 1.00 | 1.00 | 1.00 |
| SCCCRZ1001G10.g | transcription factor | hormone-related/Aux/IAA | IAA16 | 1.00 | 1.00 | 1.00 | 1.00 | 1.00 | 1.00 |
| SCCCRT1001E01.g | hormone biosynthesis | jasmonic acid | Lipoxygenase | 1.00 | 1.00 | 1.00 | 1.00 | 1.00 | 1.00 |
| SCBFSB1046D04.g* | protein kinase | SNF-like kinase | ScCIPK-16 | 1.00 | 1.00 | 1.00 | - | - | - |
| SCMCRT2103B04.g* | protein kinase | SNF-like kinase | ScCIPK-21 | 0.69 | 1.00 | 0.99 | - | - | - |
| SCCCLR1C05B07.g | protein kinase | SNF-like kinase | ScCIPK-3 | 1.00 | 1.00 | 0.99 | 1.00 | 1.00 | 1.00 |
| SCEPRZ1009C10.g | protein kinase | SNF-like kinase | ScOSA PK-1 | 1.00 | 1.00 | 0.53 | 1.00 | 0.51 | 1.00 |
| SCACLR2007G02.g | protein kinase | SNF-like kinase | ScPKABA1-1 | 0.9 | 1.00 | 1.00 | 1.00 | 1.00 | 1.00 |
| SCRFLR1034G06.g | protein kinase | SNF-like kinase | ScPKABA1-3 | 1.00 | 1.00 | 1.00 | 1.00 | 1.00 | 1.00 |
| SCEPRZ1010E06.g | protein phosphatase | serine/threonine PPM family | PP2C-like | 1.00 | 1.00 | 1.00 | 1.00 | 1.00 | 1.00 |
| SCQGLR1085F11.g | stress | drought-induced | Dehydrin | 1.00 | 1.00 | 1.00 | 1.00 | 1.00 | 0.98 |
| SCCCLR1024E11.g | stress | superoxide dismutase | Cu/Zn (SOD2) | 1.00 | 1.00 | 0.97 | 1.00 | 1.00 | 1.00 |
| SCCCRZ1001H05.g | transcription factor | HLH (helix-loop-helix) | ScbHLH7 | 1.00 | 1.00 | 1.00 | 1.00 | 1.00 | 1.00 |
| SCAGLR1021G10.g | transcription factor | homeobox | ScHB2 | 1.00 | 0.95 | 1.00 | 1.00 | 1.00 | 1.00 |
| SCCCLR2003E10.g | transcription factor | NAM (no apical meristem) | ScNAC27 | 1.00 | 1.00 | 1.00 | 1.00 | 1.00 | 1.00 |
| SCEZHR1088E02.g | protein phosphatase | tyrosine phosphatase | Dual Specificity Protein Phosphatases (DSPP) | 1.00 | 1.00 | 1.00 | 1.00 | 1.00 | 1.00 |

* : Sucrose – specific, do not respond to glucose
