# Supplementary figures and images for: Sugarcane genes associated with sucrose content
Source: BMC Genomics. 2009 Mar 21;10:120. doi: 10.1186/1471-2164-10-120 (PMC2666766; doi:10.1186/1471-2164-10-120)

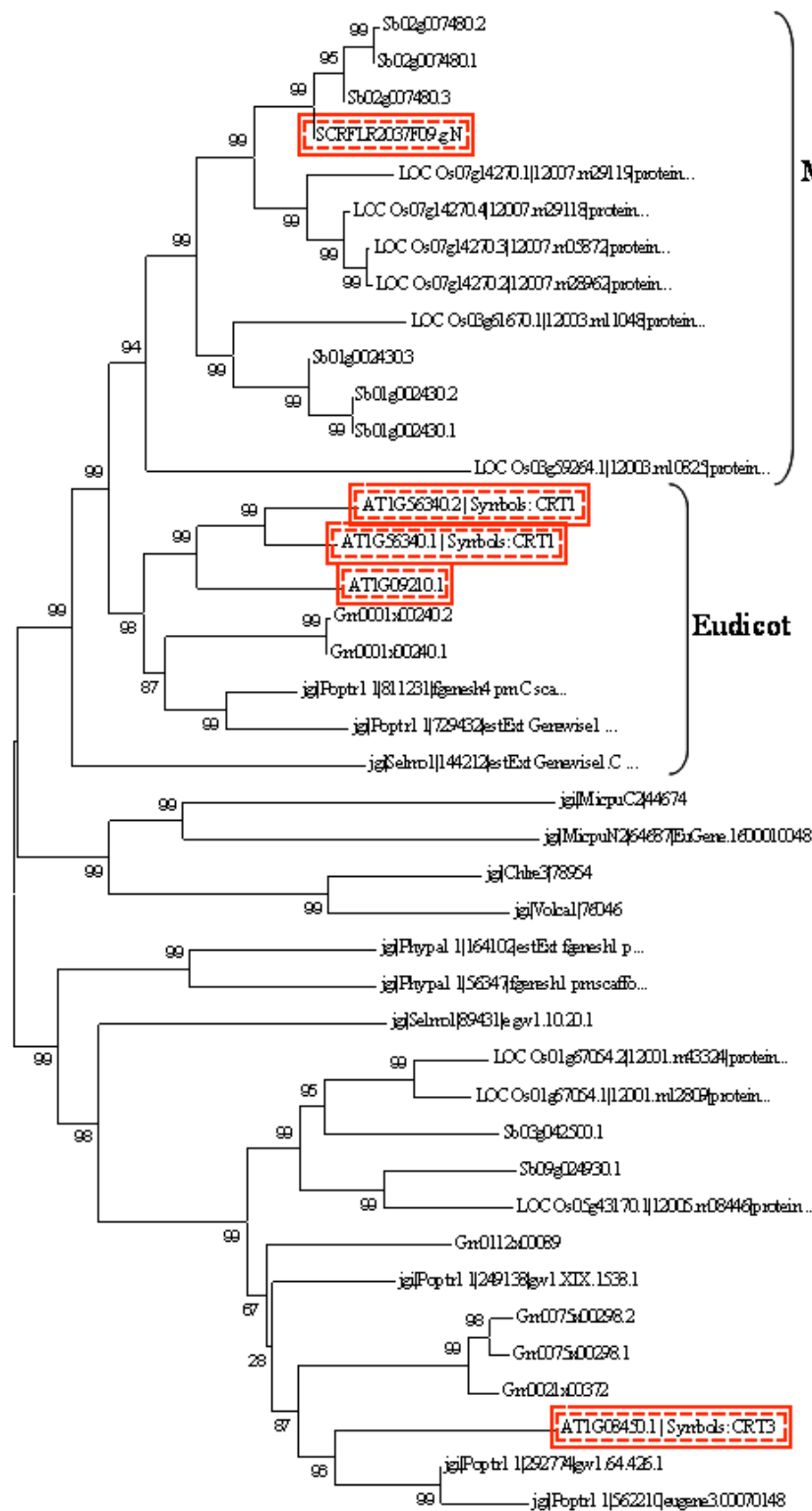

A

0.05

B

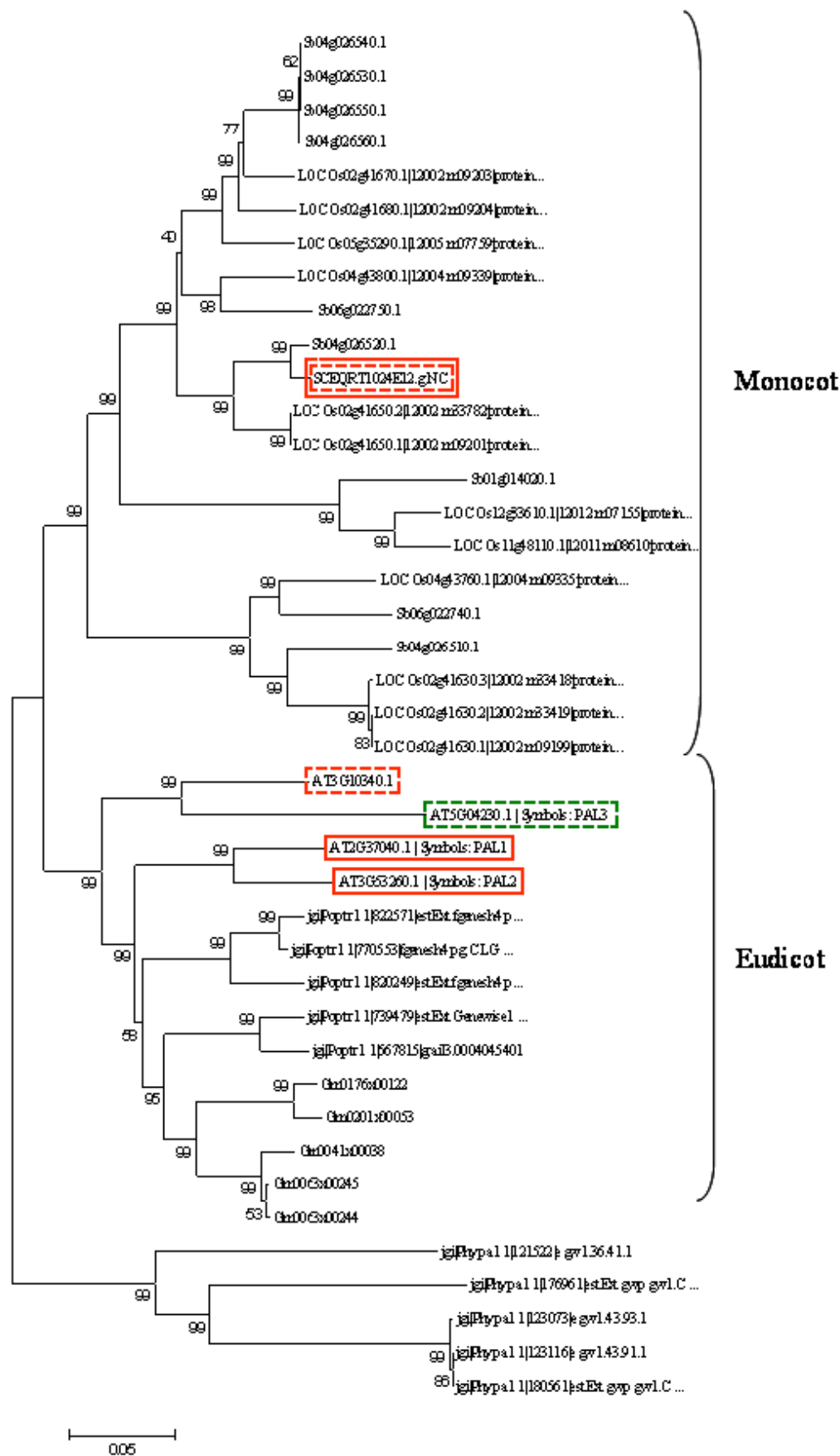

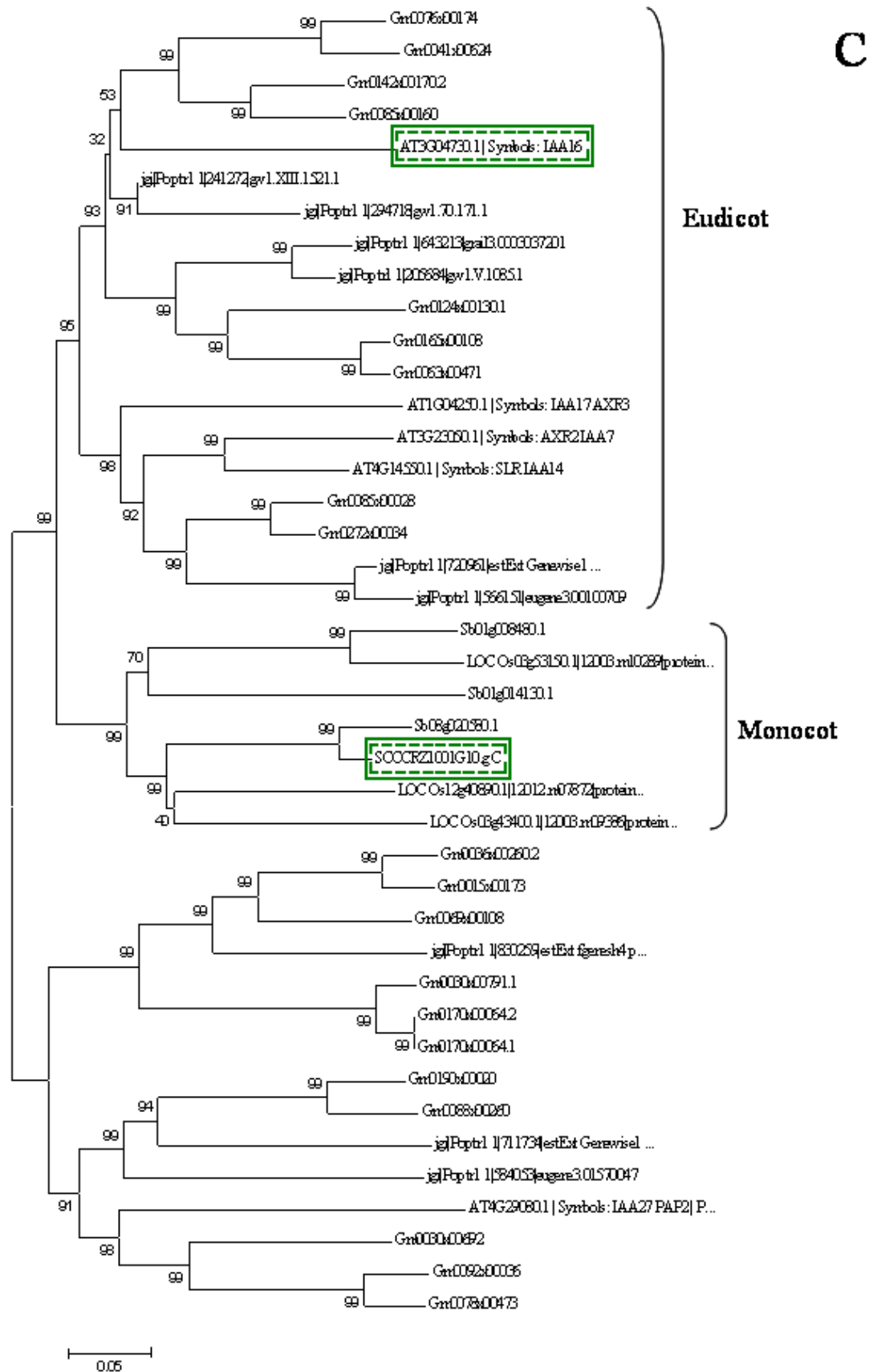

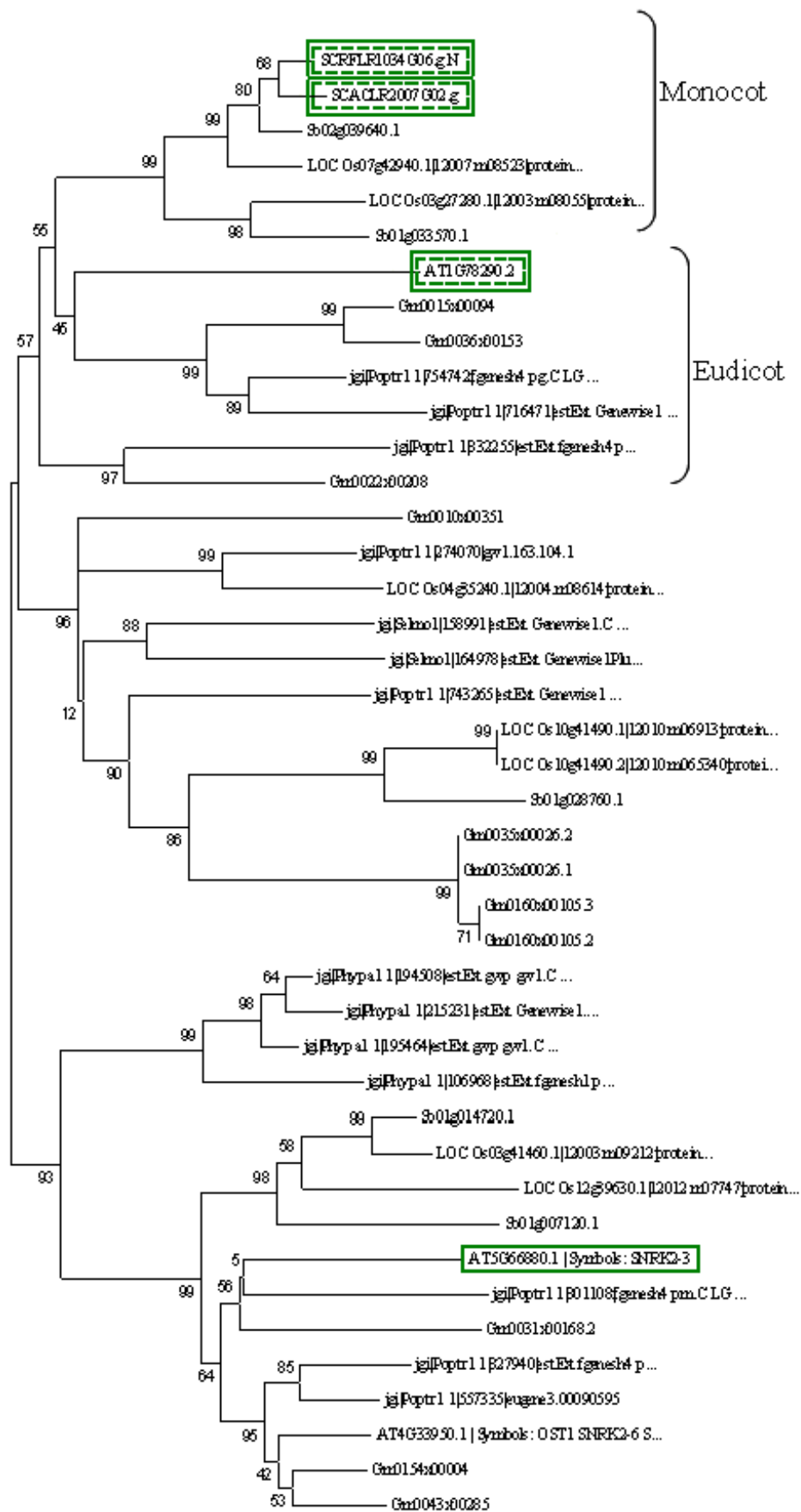

D

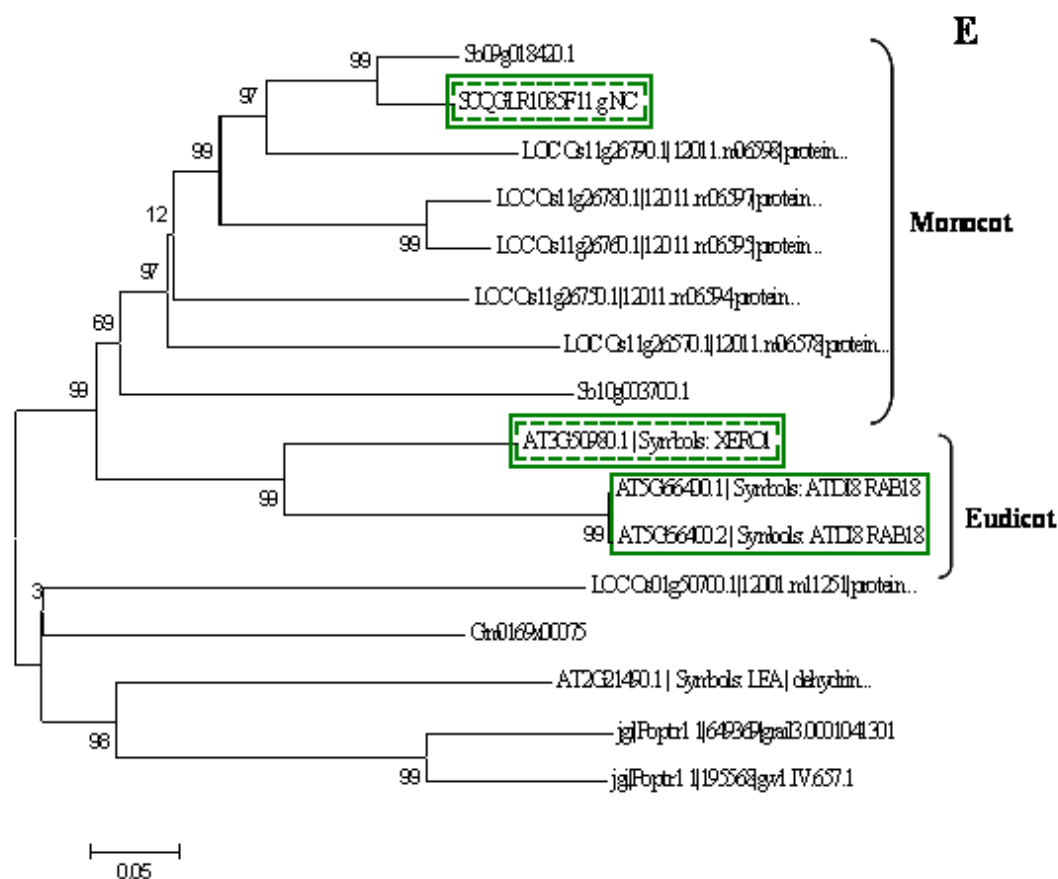

Supplement: Additional file 5 — Inferred phylogenetic relationships among tblastx hits using the sugarcane SAS as queries. The amino acid alignments were performed with ClustalX. The distances were obtained by p-distance and topography inferred with Neighbor-Joining (NJ) using only the aligned blocks (complete deletion). Analysis were conducted in MEGA4. The continuous blocks show regulation by sucrose and the pointed blocks show regulation by glucose (in both cases red for induction and green for repression). A – SCRFLR2037F09.g (Calreticulin 2); B – SCEQRT1024E12.g (Phenylalanine ammonia-lyase); C – SCCCRZ1001G10.g (IAA16); D – SCACLR2007G02.g and SCRFLR1034G06.g (canePKABA1-1 and canePKABA1-3); E – SCQGLR1085F11.g (Dehydrin). The sequences names correspond to those present in the protein data sets showed in Material & Methods: AT – Arabidopsis thaliana; Gm – Glycine max (soybean); jgi|Poptr1 – Populus trichocarpa; LOC Os – Oryza sativa (rice); Sb – Sorghum bicolor (sorghum); jgi|Selmo1 – Selaginella moellendorffii; jgi|Phypa1_1 – Physcomitrella patens patens; jgi|MicpuC2 – Micromonas pusilla CCMP1545, jgi|MicpuN2 – Micromonas strain RCC299; jgi|Volca1 – Volvox carteri; jgi|Chlre3 – Chlamydomonas reinhardtii. [file 1471-2164-10-120-S5.pdf]
